# Crossmodal correspondences between basic tastes and visual design features: A narrative historical review

**DOI:** 10.1177/20416695221127325

**Published:** 2022-10-11

**Authors:** Byron P. Lee, Charles Spence

**Affiliations:** Crossmodal Research Laboratory, 6396 University of Oxford, UK

**Keywords:** crossmodal correspondences, taste, color, curvilinearity, object mediation, emotional mediation

## Abstract

People tend to associate abstract visual features with basic taste qualities. This narrative historical review critically evaluates the literature on these associations, often referred to as crossmodal correspondences, between basic tastes and visual design features such as color hue and shape curvilinearity. The patterns, discrepancies, and evolution in the development of the research are highlighted while the mappings that have been reported to date are summarized. The review also reflects on issues of cross-cultural validity and deviations in the matching patterns that are observed when correspondences are assessed with actual tastants versus with verbal stimuli. The various theories that have been proposed to account for different classes of crossmodal correspondence are discussed, among which the statistical and affective (or emotional-mediation) accounts currently appear most promising. Several critical research questions for the future are presented to address the gaps that have been identified in the literature and help validate the popular theories on the origin and operations of visual-taste correspondences.

People display a sometimes-surprising tendency to connect various features, attributes, or dimensions of experience across the senses (Spence, 2011). While the terminology has varied over the years, the term *crossmodal correspondence* is now commonly used in the literature to identify this specific type of systematic crossmodal association (see Spence, 2011, for a review). Basic tastes, in the form of gustatory qualities (such as bitter, sour, salty, and sweet), constitute one of the often-studied attributes in the literature on crossmodal correspondences. In recent years, the associations between basic tastes and various visual features have received considerable research interest among the wide range of crossmodal correspondences that have been documented to date (Spence & Levitan, 2022; Spence & Ngo, 2012). One notable example is the consistent matching of the color green and sour taste when people are instructed to associate taste qualities with colors (Spence et al., 2015). Such correspondences also appear to be bi-directional, with the same pattern of crossmodal associations typically being reported when matching to either gustatory or visual stimuli.

This interest in, and inquisitiveness about, taste (and flavor) correspondences has often been driven by a combination of artistic curiosity and commercial incentives, especially in the case of the relationship between tastes and visual features, such as shapes and colors (Mick, 1986; Nelson & Hitchon, 1999). For instance, by making appropriate reference to these connections, marketers working with the food and beverage industry have wondered whether they might be able to develop more persuasive product presentations (e.g., Piqueras-Fiszman et al., 2012; Spence, 2021a; Spence & Youssef, 2019; Stewart & Goss, 2013). The core idea here is to try and create designs that are congruent with consumer expectations, which can hence be processed more fluently, and will thus likely lead to a more positive impression of the product (product experience) as a whole (Vogel et al., 2021). Even now, the potential application to real-life marketing design represents a potent driving force behind the majority of such research on the crossmodal correspondences involving visual features. Studies from different perspectives have emerged discussing the designs and implications that might be inspired, or enhanced, by knowledge of such crossmodal vision-taste associations (e.g., Rolschau et al., 2020; Sugimori & Kawasaki, 2022).

To date, several forms of crossmodal correspondence have been documented between basic taste qualities and visual attributes/features such as colors, shapes, and even visually-presented textures. Here, and throughout this review, the *basic taste qualities* that will be discussed include bitter, sweet, salty, sour, and umami (Duffy et al., 2009; Ikeda, 1909/2002; Kurihara, 2015), although many studies have focused on only a subset of these qualities (Cecchini et al., 2019). Visual-taste correspondences involve the systematic mapping of the basic tastes to one or more dimensions of visual perception. Notably, form-taste (also referred to as shape symbolism or shape-taste crossmodal correspondences) and hue-taste (also referred to as color symbolism or color-taste correspondences) constitute the earliest and most widely studied types (see Spence, 2012; Spence & Levitan, 2021; Spence & Ngo, 2012, for reviews of the color-taste and shape-taste correspondences). Over the past few decades, there has been a rapid development in the published literature on crossmodal correspondences between basic tastes and these two visual attributes. While early studies tended to focus on the within-individual consistency of color-taste matching (e.g., Déribéré, 1978; O’Mahony, 1983), the research that has been published in recent years has gradually shifted to focus on the consensuality of such mappings and people's metacognitive awareness when making their inferences concerning the most appropriate match (e.g., Koch & Koch, 2003; Spence & Levitan, 2021). More recently, studies have started to incorporate online testing methods and to assess the influence of cross-cultural factors on crossmodal correspondences (Spence, 2022; Spence et al., 2015; Velasco et al., 2016b; Wan et al., 2014b; Woods et al., 2013). 

Crossmodal correspondences involving gustatory qualities can take place at both a conceptual and perceptual level. People would choose similarly when tasked with connecting the verbal concepts of different senses, as in pairing the words of taste with that of a visual feature (e.g., people typically report that the word “red” matches the word “sweet” and vice versa; O’Mahony, 1983). At the same time, however, these correspondences have also been observed at a perceptual level, such as between color patches and actual tastants (e.g., Saluja & Stevenson, 2018; Velasco et al., 2015b; Velasco et al., 2016a). When manipulated appropriately, the presentation of visual stimuli has even been shown to modify people's expectations and perception of gustatory stimuli (e.g., Liang et al., 2016; Stewart & Goss, 2013; Velasco et al., 2018a). The latest developments in this literature have tended to focus on analyzing the validity of those theories that account for relevant correspondences (e.g., Higgins & Hayes, 2019; Whiteford et al., 2018), that have identified several factors potentially mediating crossmodal associations (Spence & Levitan, 2021). While the understanding of taste-visual crossmodal correspondences has seen substantial progress in recent decades, there remain a number of important questions that have yet to be addressed.

This narrative historical review therefore identifies critical gaps in the academic literature and suggests promising directions for follow-up studies. The review begins with a summary of the documented correspondence between basic taste qualities and visual features, which details the bi-directional matches of taste with color and taste with shape. Then, with reference to the empirical evidence, the prospect of translating associations into designs that may influence taste evaluations and perception is assessed. The final part of the review examines prominent theories concerning the cognitive mechanisms underlying crossmodal correspondences.

## Crossmodal Correspondences Between Taste and Visual Features

Over the past few decades, the literature on visual-taste crossmodal correspondences has nearly always analyzed specific visual features, such as color and shape, while distinguishing basic taste qualities (Spence et al., 2015; Velasco et al., 2014). Occasionally, attempts have also been made to assess those correspondences that people have with taste intensity (Saluja & Stevenson, 2018; Wang et al., 2016). These approaches have led to the now well-documented cases of color-taste and shape-taste correspondences. Among them, shape-taste mappings have been broken down into curvilinearity-taste and symmetry-taste correspondences (Salgado-Montejo et al., 2015).

The processing of color and shape takes place in parallel in the visual system (Desimone et al., 1985; Goodale et al., 1994; Pickford, 1948). In fact, the neurophysiological evidence shows that the processing of visual information is more specialized and occupies a far greater part of the neocortex than does the processing of gustatory information (Felleman & Rakic, 1991). In contrast, gustatory information processing is primarily concerned with identifying the taste quality and determining the intensity of the signal (Carleton et al., 2010). Given that humans are visually-dominant (Hutmacher, 2019), one might reasonably expect that associations with visual features would be the most frequently investigated types of crossmodal correspondences with taste.

The following sections summarize previous attempts to document the crossmodal associations that have been established between taste qualities (and taste intensity) and specific visual features. Where such information is available, the cross-cultural consensuality of the association patterns will also be discussed. By building up the case with relevant evidence and highlighting some of the key gaps in the academic literature, this chapter provides an opportunity to systematically reflect on the progress that has been made in the field to date.

## Crossmodal Correspondences Between Basic Tastes and Colors

Color (specifically, hue) is one of the most widely-studied visual features in the literature on crossmodal correspondences that has been published to date, offering arguably the most “natural” link to basic taste (Wan et al., 2014a). With color effectively indicating various food properties, people have learnt to rely on color cues to identify and evaluate food through millennia of foraging activities (Lieberman, 2006). The last few decades have seen a wealth of research dedicated to mapping out the crossmodal associations between color and basic taste qualities, making use of a variety of experimental approaches to assess how people match color with taste. In its most primitive form, a possible system of color-taste associations was first entertained by the marketing and advertising industry to help promote powerful communication in visual designs (e.g., Favre & November, 1979). With fewer theoretical frameworks to work with, these early discussions would appear to have been inspired by people's intuitions, with seemingly little to no empirical data (or at least none made publicly available) to support the claims. Favre and November’s (1979, p. 31) book on color marketing, for instance, lacked any citations to underpinning scientific research (as highlighted by Ernst, 1980). Despite the critics, the mappings documented by these early studies have been shown to be consistent with many of the later findings obtained over the subsequent decades (see Spence & Levitan, 2021).

One of the earliest studies to have documented color-taste correspondences was conducted by the French chemical engineer Déribéré (1978) in relation to his discussion of the connections between perfume and color. At one point, Déribéré casually reports the colors most frequently associated with the four basic tastes (see Table 1). As the study was of a supplementary nature, little information was provided about the methodologies used to obtain these matches (nor the sample sizes involved). The relationship between tastes and colors was not examined in the paper, as Déribéré chose not to comment on his results and made no speculations about the origin of the correspondences.

**Table 1.** The Color Associations of Four Basic Tastes Established in Different Studies.

Study	Sweet	Sour	Salty	Bitter
Déribéré (1978) ^a^	Red White	Yellow Green	Blue White	Brown Green Blue
Favre & November (1979) ^b^	Orange Pink Red	Yellow Green	Gray Pale Green Pale Blue	Blue Brown Green Violet
O’Mahony (1983)	Red	Yellow	White	-
Koch & Koch (2003)	Red Orange	Yellow Green	White	-
Wan et al. (2014b)	Pink	Green	White	Black
Raevskiy et al. (2022) ^c^	Pink Light Red	Yellow Green	White Blue	Dark Green Brown

*Note.* Dash (-) denotes no color associations found for the given taste.

^a^ Closest hue category based on the names used in Déribéré’s (1978) questionnaire.

^b^ Approximate hue category based on the color schemes recommended.

^c^ Approximate hue category based on the color panel presented to the participants.

Among the pioneering attempts to document color-taste matches, a much more comprehensive study was reported by O’Mahony (1983), in which students (*N* = 51) were tasked with picking one from a list of 12 common color terms (red, orange, yellow, green, blue, violet, brown, black, white, gray, silver, and gold) “to describe” each of the four basic tastes. These tasks were carried out three times, separated by two-week intervals. Critically, unlike most of the later studies, the method used by O’Mahony involved assessing within-participant consistency by determining the proportion of the participants who consistently picked the same color to match with a given taste each time they were asked. This approach may well have excluded those cases in which a participant considered a taste as matching with more than one color. Note also that such a closed responding design would have restricted the discovery of color matches to the set of hues selected by the researcher.

Curious about how color appearance might affect taste perception, Koch and Koch (2003) developed a survey to examine the associations that participants held between ten common color words (red, green, yellow, blue, brown, orange, purple, black, gray, and white) and four basic taste qualities. The participants (*N* = 45) had to rate the intensity of every basic taste quality for each color with questions such as “how sweet is the color red?” Contrasting with O’Mahony’s (1983) study, in which the researcher collected votes of the single most strongly associated color for a given taste, Koch and Koch assessed the strength of associations between the colors and tastes. Although still limited to the options presented by the researchers, this approach revealed some of the color-taste associations previously overshadowed by more dominant colors. For example, red was regarded as the only “sweet color” in O’Mahony's study because their participants could only pick one color, but Koch and Koch found orange to be another significant match for sweet (see Table 1).

Even though the approach used by O’Mahony (1983) only recorded the most frequently selected color for each taste quality when compared to Koch and Koch’s (2003) or Déribéré’s (1978) findings, the three studies nevertheless still exhibit a high degree of consistency among the established color-taste pairs (see Table 1). Putting these findings together, it would appear that people find bitter to be the most ambiguous taste to match with a particular color. According to the results of O’Mahony's frequency analysis, only a small number of the participants (12 out of 51) consistently associated bitter with a specific color, of which no color stood out as a consensual choice. Similarly, in Koch and Koch's chi-square analysis, although there was a statistically significant association between bitter and black, the authors did not consider the relationship to be a reliable one. Such ambiguity could partially be attributed to the confusion surrounding the taste labels of bitter and sour in some individuals (e.g., O’Mahony et al., 1979). As discussed in the later parts of this review, some recent studies have also discovered a similar trend when testing the color correspondences of bitterness in participants from more diverse cultural backgrounds (Raevskiy et al., 2022) and with real tastants (i.e., rather than with taste descriptors; see Sugimori & Kawasaki, 2022).

Some common limitations can be identified when looking back at the early visual-taste crossmodal correspondences experiments, shared by the studies covered thus far (and also by the majority of the other early research). For example, instead of using actual color stimuli, such as a palette or a swatch chart of colors, the researchers presented verbal descriptions of colors in their association tasks. On the other side of the correspondence, the concept of taste qualities was typically also represented by the words describing the basic tastes (i.e., standing in for actual gustatory stimuli). Using the results of such research to support the existence of specific color-taste correspondences would require the assumption that lexical representations do indeed provide a reasonable approximation of sensory stimuli. This question was also raised in Simner et al.’s (2010) *a posteriori* analysis on the usage of verbal labels when matching crossmodal concepts, in which they speculated that the use of linguistic stimuli may have exerted an unanticipated influence over mappings when compared to mappings between sensory stimuli.

Another potential limitation of these early studies is relying on participants from a restrictive demographic and therefore assuming the data collected to be representative of the population at large. With college students being the only source of recruitment, the findings in the studies discussed so far (e.g., Koch & Koch, 2003; O’Mahony, 1983) were restricted to a very specific demographic, one that is nowadays often described as *WEIRD* (i.e., people from Western, Educated, Industrialized, Rich, and Democratic backgrounds; Henrich et al., 2010). Although the lack of a broader representation has not been cited as a criticism of the validity of the research, it is commonly maintained that many types of crossmodal correspondence are subject to a certain degree of cultural variations (see Liang et al., 2016; Chen et al., 2016; Raevskiy et al., 2022).

Wan et al. (2014b) organized a large-scale online study to address the potential biases produced from over-sampling WEIRD populations. Wan et al. (2014b) tested crossmodal correspondences involving the five basic tastes (including umami/savory) and 11 color patches (black, blue, brown, green, gray, orange, pink, purple, red, white, and yellow). 428 participants were recruited from four countries (China, *n* = 144; India, *n* = 113; United States, *n* = 117; and Malaysia, *n* = 54) of distinctively different cultural backgrounds, and ranged from 17 to 66 years of age. The results revealed a similar pattern of color-taste correspondences across different cultural groups, with a high degree of consistency when compared to the previous findings (i.e., Koch & Koch, 2003; O’Mahony, 1983). For instance, the participants matched pink with sweet, green with sour, white with salty, and bitter with black. Some minor discrepancies were also observed in terms of specific matches. So, for example, the matching of yellow with sour was noticeably missing among the Indian participants, while the white-salty match was reported significantly less often by the participants who were from mainland China (see also Liang et al., 2016).

Recently, Raevskiy et al. (2022) extended the study of such cultural similarities and differences in color-taste associations to individuals (*N* = 338) from a range of non-Western backgrounds. They found the color associations of sweet, salty, and sour to show a high degree of consistency among participants from Japan (*n* = 136), Russia (*n* = 102), and Taiwan (*n* = 100), following the pattern established by the prior research (see Table 1). However, the color representations of bitter taste appeared to vary somewhat from one culture to the next (although a consensus was formed around dark green and brown), which happens to be in line with the ambiguity of bitterness reported by O’Mahony (1983) and Koch and Koch (2003). The most drastic difference was obtained between the Russian and East Asian participants when matching colors with the taste of umami; the patterns of lower consensuality and higher diversity in the results reported by the Russian participants seem to be caused by the lack of knowledge and familiarity in their culture. Curiously, yellow was reported as a color that was associated with the taste of umami by the Japanese participants, agreeing with the Ikeda’s (1909/2002) intuitions when he first scientifically reported on the existence of umami more than a century ago (though Brillat-Savarin, 1835, described something very similar almost 70 years earlier).

This pattern of cultural universality with occasional minor discrepancies has also been documented in other areas of crossmodal correspondences research. For instance, Jacquot et al. (2016) explored cultural differences between the UK (*n* = 59) and France (*n* = 60) regarding odor-color correspondences. Of the 16 odors that they tested, three were found to be associated with different colors by the two populations. Here, though, it should be noted that even among the odors matched differently between the two cultures, the disagreements were fairly subtle (such as blue-green in the UK vs. green in France, or pale yellow in the UK vs. orange in France).

Contrary to the paradigms used by O’Mahony (1983) and Koch and Koch (2003), Wan et al. (2014b) used color patches as stimuli rather than color words in the association task. Essentially, in addition to demonstrating cross-cultural consistency in color-taste crossmodal correspondences, Wan et al. (2014b)’s findings also support the feasibility of using color words in taste association research, as both color labels and the actual color patches appeared to have yielded a generally consistent mapping of taste to color. Although the color patch and color word gave rise to an identical pattern of taste associations in Wan, Woods et al.'s study, it is unknown if they were driven by the same underlying mechanism. As suggested recently by Pedović and Stosić (2018), taste information conveyed by the visual sensory stimuli could perhaps be induced and mediated by different factors than were conveyed by the word stimuli. The factors that might mediate the operation of visual-taste correspondences will be further discussed later in this review.

## Crossmodal Correspondences Between Taste and Shape

Similar to the crossmodal correspondences that have been documented between color and taste, a wealth of empirical research now demonstrates the existence of crossmodal correspondences between abstract shapes and basic tastes (Spence & Ngo, 2012). What is more, interest in understanding the mapping of basic taste and shape can once again be traced back to food marketing, particularly the desire to be able to predict, or perhaps even to manipulate, people's taste expectations using shape (Cheskin, 1967; Dichter, 1971; see Chitturi et al., 2019, for a contemporary example). In the relevant literature, shape is typically reduced to a handful of basic components; notable examples are curvilinearity (roundedness/angularity), symmetry, shape weight (thinness/thickness), and segment complexity. As summarized by Velasco et al. (2016c), the majority of shape-taste studies that have been published to date have chosen to focus on curvilinearity. Among these studies, a consensus has been reached on the mapping between curvilinearity and the five basic tastes. People typically associate rounded shapes with sweet taste, while associating angular shapes with sour, salty, bitter, and umami tastes.

The idea of pairing sweet taste with rounded shapes and bitter taste with angular shapes has been floating around in the literature for more than half a century now (Dichter, 1971; see Spence & Ngo, 2012, for a review). That being said, there have been far fewer studies on the taste-shape correspondences than on taste-color correspondences (Velasco et al., 2016c). Marks (1978, pp. 186–191) commented on the similarity of tasting and touching, noting how the linguistic history of certain adjectives (e.g., “sharp”) had shifted over time from first describing touch to later taste and now visual shapes. Relevant to the affective account, a theory that places emotion as the key mediator between the crossmodal connections, Marks (1978, p. 75) even suggested hedonic value to be responsible for the qualitative similarity between intrinsically pleasant stimuli. According to the affective account, this similarity would, in turn, motivate people to associate properties of a similar hedonic value, such as sweet taste and rounded shapes.

The first study to have assessed the putative links between shapes and tastes was essentially an exploratory investigation on the *synesthesia* of “tasting shapes” (Cytowic & Wood, 1982). Synesthesia refers to the involuntary and automatic sensory experience (i.e., concurrent) that is sometimes perceived in one sensory modality in response to the stimulation (an inducer) in another modality (Cuskley et al., 2019; although see Simner et al., 2005, for the intramodal synesthesia between graphemes and colors in synesthetes). The study conducted by Cytowic and Wood focused on the experience of geometric shape concurrents experienced by a synesthete when exposed to a gustatory (inducing) stimulus. They also collected the associations reported by three neurotypical individuals (i.e., non-synesthetes) in their shape-taste matching survey. While such a small sample size is, of course, unlikely to deliver crossmodal mappings that are in any sense statistically meaningful, the study nevertheless represents what we believe to be the first published attempt to document how non-synesthetic individuals would choose to associate taste with geometric shapes. Because the shape stimuli used by Cytowic and Wood were developed on the idiosyncratic intuitions of the study's one synesthetic participant, the results cannot be interpreted as providing a conventional match between tastes and specific geometric features. That said, some interesting patterns can nevertheless still be observed: For instance, the three non-synesthetes all picked a sphere-like shape to match the sweetest taste; They also picked a pointy pyramid (e.g., triangular, square, or pentagonal pyramid) to match the sourest taste (see Table 2).

**Table 2.** Shape Features Associated with Each of the Basic Tastes.

Study	Taste stimuli	Taste quality			
		Sweet	Sour	Salty	Bitter
Cytowic & Wood (1982) ^a^	Solution	Sphere	Pyramid		
Ngo et al. (2011)	Chocolate				Angular
Spence & Ngo (2012)	Beer	Rounded, Voluminous			Angular, Thin
Salgado-Montejo et al. (2015)	Word	Symmetry, Simplicity			Angular
Velasco et al., (2015a)	Word	Rounded	Angular	Angular	Angular
	Solution	Rounded	−	−	−
Velasco et al., (2016a)	Solution (Weak)	Rounded	Angular	Angular	−
	Solution (Strong)	Rounded	Angular	−	−
Turoman et al. (2018)	Word	Symmetry	Asymmetric		Asymmetric

*Note.* Dash (-) denotes links that were assessed but no significant effect was found to suggest a meaningful association.

^a^ The original data were insufficient for statistical analyses; the associations listed here are those observed to be a noticeable trend.

**Figure 1.**
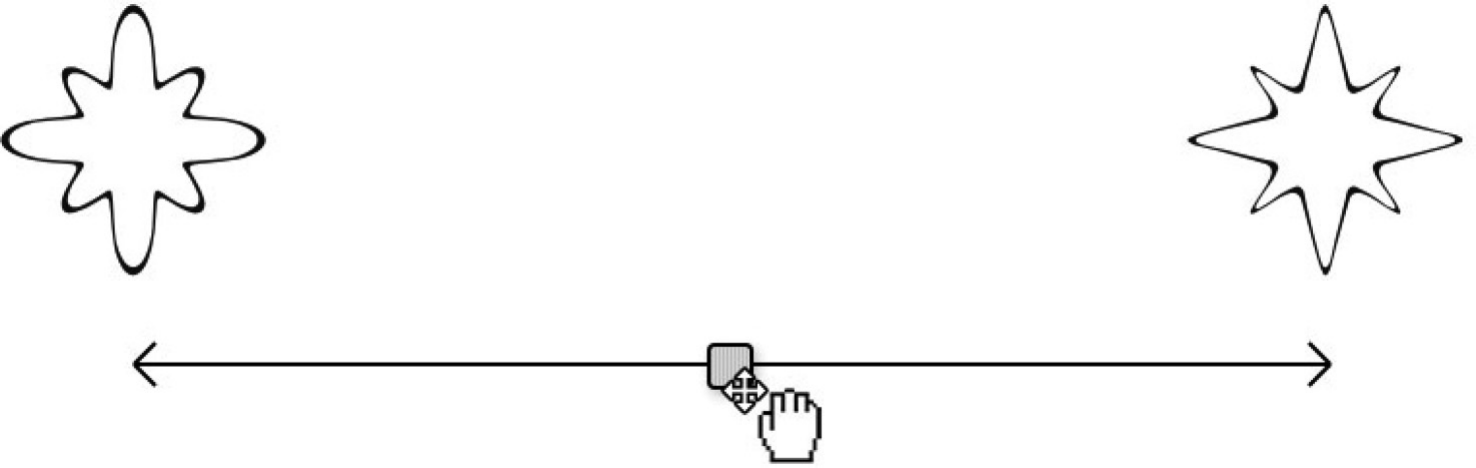
A mock-up of the visual analogue scale (VAS) used to collect the degree of shape curvilinearity.

Among the later studies that have examined the crossmodal mappings between basic taste and shape with a more systematic approach, the pairings of rounded shapes with sweet and angular shapes with other tastes (sour, salty, bitter, and in the relevant cases, umami) have been observed consistently (e.g., Deroy & Valentin, 2011; Ngo et al., 2011; Velasco et al., 2015a). In general, these studies have adopted a similar design to probe the crossmodal association between shape and taste: The participants were asked to find a position for the presented taste stimulus on a Visual Analogue Scale (VAS; see Figure 1) or a Likert scale. The two ends of this scale were anchored by an angular shape and a rounded shape, respectively.

**Figure 2.**
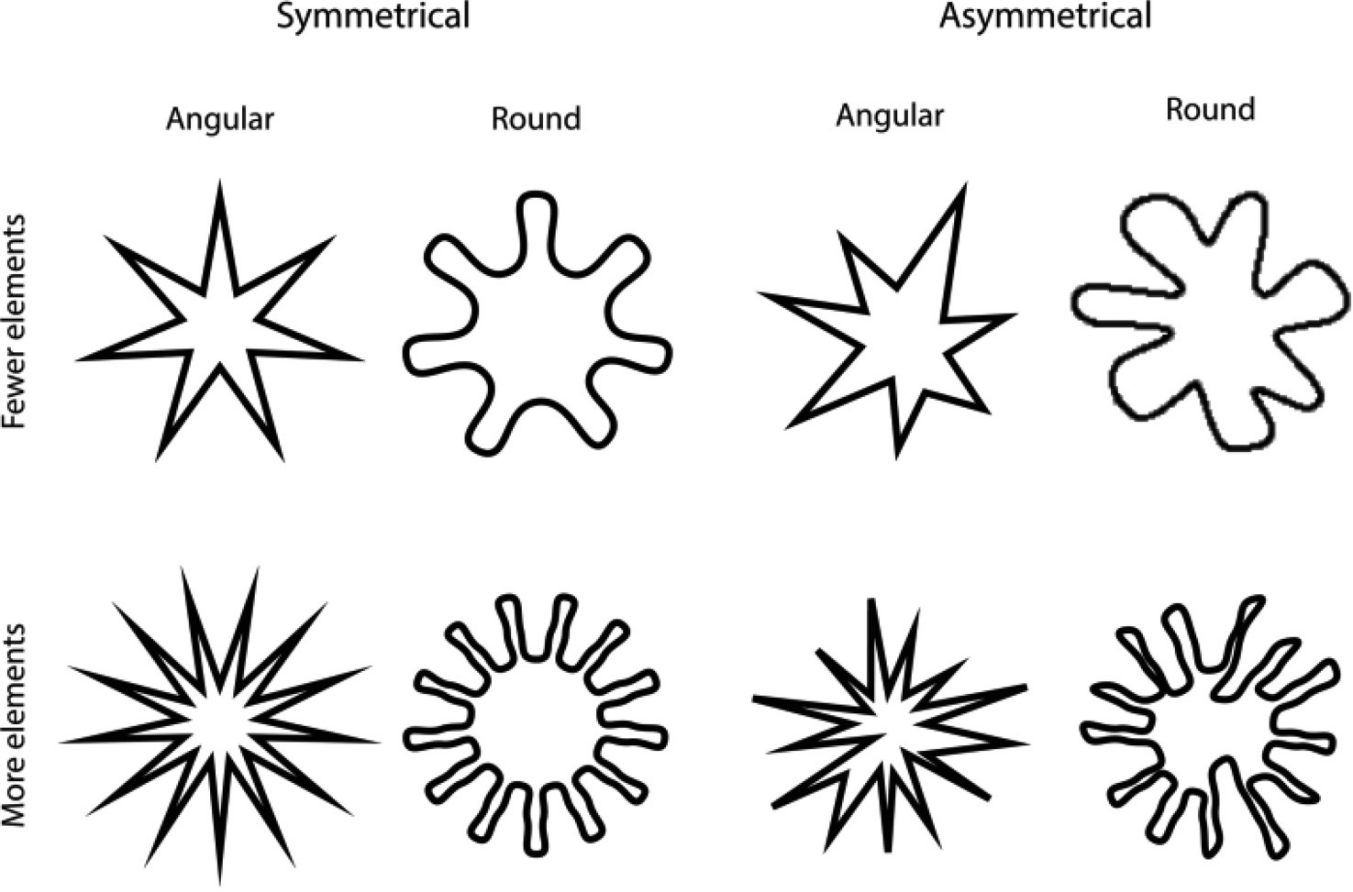
The visual stimuli varying in terms of three shape features: curvilinearity, symmetry/asymmetry, and segment complexity.

*Note*. Adapted from “The sweetest thing: the influence of angularity, symmetry, and the number of elements on shape-valence and shape-taste matches,” by Salgado-Montejo, A., Alvarado, J. A., Velasco, C., Salgado, C. J., Hasse, K., & Spence, C, 2015, *Frontiers in Psychology, 6*. CC BY 4.0.

Up to this point, most studies on shape-taste correspondences have chosen to focus on the contour angularity of shape, otherwise known as curvilinearity, while other geometric features have remained relatively untouched. After analyzing several geometric features and their prospect of being associated with positive emotion, Salgado-Montejo et al. (2015) introduced the concept of visual symmetry/asymmetry into their experiment. Salgado-Montejo et al. formulated their hypothesis based on the previous findings that shapes with symmetric characteristics are typically rated as pleasant (see Makin et al., 2012) and more likely to be associated with tastes of similar valences, such as sweet and umami. For their study, Salgado-Montejo et al. designed a series of shapes varying not only in curvilinearity but also in symmetry and segment complexity (see Figure 2). Additionally, participants were recruited from the United Kingdom (*n* = 15) and Colombia (*n* = 18) to identify any cross-cultural differences between these two countries. The symmetry and segment complexity of shape was found to exert a considerable influence over participants’ associations with taste. In both countries, symmetrical shapes and shapes with fewer elements were more consistently matched with a sweet taste. A significant interaction was also observed between symmetry and fewer elements, which led to sweeter ratings in both countries, indicating synergy between the different visual attributes when conveying taste information.

Following the confirmation of visual symmetry-taste correspondences, Turoman et al. (2018; *N* = 90) went on to investigate how specific types of shape symmetry would influence the pattern of shape-taste associations. They demonstrated that shapes with both reflectional and rotational symmetry were rated as more pleasant and found to be most strongly associated with the taste of sweetness, followed, in decreasing order, by shapes with only bilateral symmetry and asymmetric shapes; Sour and bitter were associated with these features in precisely the opposite manner. Critically for the affective theory, Turoman et al.'s research has also contributed an evolutionary account to the explanations of the underlying mechanisms of crossmodal correspondences (Salgado-Montejo et al., 2015). The correlation between sweetness and pleasantness ratings found in their study was in line with the prediction of a preference-driven association between symmetrical features and sweet taste. However, as Turoman and their colleagues noted, their findings should not be taken as providing definitive evidence of a causal link between valence evaluation and taste association.

While all basic taste words were found to have a robust match with either rounded or angular shapes (e.g., Deroy & Valentin, 2011; Spence & Ngo, 2012), there appears to be a noticeable discrepancy in terms of the mappings when the participants actually tasted the stimuli. The contrast between taste words and real tastants was highlighted by Velasco et al.’s (2015a) study, in which the shape association of taste words and real tastants was measured using a VAS angularity scale. Like other reports, when using taste words as stimuli, the participants consistently matched tastes with shapes following the previously-established pattern. However, with real tastants (*n* = 12), the observed patterns in their results were somewhat different. In particular, Velasco et al. reported that the sweet-tasting solution was matched with rounded shapes, but no significant association was documented for the other basic tastes (sour, salty, bitter, and umami).

Subsequently, Velasco et al. (2016a) conducted a similar study with real tastants delivered at two concentrations (*N* = 19). This time, the researchers found that sour and sweet tastants were associated with angular and rounded shapes, respectively. The salty tastant was also associated with angular shapes when administered at the lower concentration, but there were no observable taste-shape associations for either the bitter or umami tastants. In an attempt to determine what other factors might influence the shape matches, Velasco and his colleagues also had their participants rate their liking for, and the perceived intensity of, each of the solutions they tasted. Their regression model demonstrated that the concentration of the tastant and liking predicted more than half of shape matches by accounting for approximately 59% of the variance in curvilinearity ratings. These results suggested the three factors (taste quality, concentration, and valence) to be significant predictors of the shape curvilinearity that was found to be associated with the given stimulus.

Looking back over the studies that have been reviewed thus far, there exists a clear trend of taste correspondences for shape features in terms of curvilinearity and symmetry. While taste word associations with shape are consistent for all basic tastes, this consistency does not fully translate when matching real tastants with shapes. Curvilinearity was less consistently matched with the non-sweet basic tastes when assessed with real tastants, especially with those solutions that were bitter (Velasco et al., 2015a; Velasco et al., 2016a). One possibility here is that taste words, especially the non-sweet tastes, are associated with a more negative valence when processed linguistically. In other words, seeing sour, salty, bitter, and umami is evaluated as a more unpleasant or undesirable experience than actually tasting them. Consequently, presenting non-sweet taste words results in stronger taste associations than physically administering the actual tastants (Marks, 1996). Another explanation holds that the heightened sensitivity to bitterness has led to the inhibition of arousal systems (Kalat & Rozin, 1973), which, in turn, may have suppressed people's tendency to look for visual associations whenever the perceived taste intensity is low (Sugimori & Kawasaki, 2022).

## Comparing Taste Words With Actual Tastants

As discussed in the section on shape-taste correspondences, the consistency of taste mappings would appear to fluctuate somewhat between those experiments that have incorporated real tastants and those that have used taste words instead. Granted that the difference by itself is not a contradiction, it can nevertheless be a cause for concern among researchers if taste words fail to offer a satisfactory measure of the shape-taste matching as the real tastants have been shown to do. Meanwhile, the studies on color-taste correspondences that have been presented so far have also used taste words to assess associations; participants in these studies did not taste any stimuli when making their color associations. Two issues can be raised here: First, associations established with verbal taste stimuli might differ from those assessed with sensory stimuli. Second, those patterns that have been established on the basis of matching taste words might not work as expected when they are used to devise designs that wish to influence taste expectation or perception (e.g., in the case of food marketing; Aoki & Akai, 2022).

In a Stroop study carried out by Razumiejczyk et al. (2015), participants (*N* = 105) had to call out the name of the fruit shown on the screen as rapidly as possible after having tasted a scoop of fruit slurry without having seen it. This task was carried out while looking at an onscreen cue word. The participants completed the task in three different conditions: The word on screen could be the name of the fruit that the participants were tasting (congruent condition), it could also be the name of a different fruit (incongruent condition), or it could be the name of an inedible object (control condition). Overall, the researchers documented shorter RTs and higher accuracy rates for name identification when the screen text happened to be congruent with the tastant. The Stroop effect found here suggested that gustatory experience would compete with visual linguistic information for attentional resources. Razumiejczyk et al. concluded that their findings provided evidence for the crossmodal integration of taste (sensory experience) and taste words (lexical information). It should, however, be noted that the integration of sensory and verbal stimuli does not guarantee the same crossmodal mapping patterns when matched with visual features.

Previously, Demattè et al. (2009) reported on a series of similar discrimination tasks, in which the participants (*N* = 41) had to make speeded identification responses to odorants while being presented with either congruent or incongruent visual stimuli. These visual stimuli either had a shape resembling the silhouette of a fruit or the color of a fruit, which reflected the smell presented in the congruent condition. As Demattè et al. expected, they found an interference effect caused by the visual distractors in the incongruent condition. One explanation offered by the researchers suggests that verbal representations were implicitly engaged in memory during the decision-making process of odor identification, the possibility of which would help to strengthen the feasibility of guiding perceptual expectations with verbal cues (Naor-Raz et al., 2003).

While the use of verbal stimuli in crossmodal correspondences research has never been seriously challenged, it remains a potential issue that could limit the effectiveness of any attempts to modify sensory perception in real life. As such, Saluja and Stevenson (2018) tested color-taste matchings using tastant solutions (i.e., instead of words referring to basic tastes as used in the majority of the previous research). The participants in their study (*N* = 50) picked a hue from the color wheel to match with a basic taste after having tasted it in solution. Their mapping of color and taste was highly consistent with the prior literature that had used verbal stimuli. In other words, the color associations made with real tastants were comparable to those made with taste words. Combined with evidence from Razumiejczyk et al.’s (2015) study, it would therefore appear safe to conclude that using taste words in crossmodal matching experiments involving taste provides a practical alternative to using real tastants.

Returning to the patterns of color-taste correspondences that were reported by O’Mahony (1983), Koch and Koch (2003), and Raevskiy et al. (2022), the associations between bitter and its matching colors were notably less consensual than for the other tastes. With reference to the habituation effect of more accessible stimuli, the ambiguity around bitterness could partially be related to the fact that the detection threshold of bitterness is the lowest among all the basic tastes (i.e., requiring the lowest concentration to be detected; McLaughlin & Margolskee, 1994). It should also be noted that there is a larger variety of bittering agents (sometimes referred to as bitterants) and a broader distribution of bitter taste receptors than for any of the other tastes (Adler et al., 2000). Perhaps, therefore, the distinctiveness of bitter taste perception may have tuned the discrimination and sensitivity of this basic taste quality in a manner that is qualitatively different from the others (Kalat & Rozin, 1973; see also Mura et al., 2018).

By asking participants (*N* = 342) to taste chocolate and green tea of varying levels of bitterness, Sugimori and Kawasaki (2022) were able to investigate the effect of taste intensity on color-taste correspondences. The food and drink were offered to participants at two different levels of bitterness (strong or weak). In terms of visual presentation, the chocolate was wrapped in either black or pink paper; green tea was filled in a translucent plastic cup having either a clear or blue tint. The participants tasted the sample stimulus and evaluated its sweetness and bitterness in each condition. Sugimori and Kawasaki's results revealed that color information modified taste perception only when the participants happened to be tasting the more bitter food/drink. For bitter chocolate, the pink wrapper resulted in the chocolate being rated as tasting sweeter, while the black wrapper resulted in it being rated as tasting more bitter instead. The bitter green tea tasted from the clear cup was rated as tasting sweeter and less bitter than when tasted from the translucent blue cup. However, when tasting chocolate and green tea that was less bitter (i.e., the weaker level), no difference was found between any color pair for either the sweet or bitter ratings. While the underlying mechanism behind these findings has yet to be confirmed, it seemed as if tasting the less bitter stimulus inhibited vision's influence over taste perception.

As far as shape-taste correspondences are concerned, although numerous studies have documented the matches between real tastants and the shapes with either angular or rounded features (e.g., Velasco et al., 2015a; Velasco et al., 2016a), there has been a notable absence of attempts to map the verbal terms of shape features to basic tastes. Understandably, there might not be much commercial interest in the taste associations of some geometric descriptions (as in spelling out the word) such as “asymmetric” and “rotational,” as they are rarely used as verbal terms in product design (or flavor descriptions). On the other hand, product presentation occasionally demands sophisticated verbal descriptions and analogies; terms like “thin acidity” and “round bodied” are commonly used by the alcohol industry to introduce their products. Moreover, investigating the link between verbal and sensory stimuli may well turn out to be a rewarding area for those wanting to understand the origin of shape-related associations. If shape-taste correspondences have such a deep root in linguistic development as has been suggested previously (Marks, 1978, pp. 186 − 191), the verbal stimuli describing shape features might behave in a similar manner to the abstract shapes.

As demonstrated by Saluja and Stevenson (2018), it would appear that the associations mapped between the lexical concepts of taste and color can provide an accurate representation in lieu of presenting actual sensory stimuli (which comes with its own practical challenges). What this means for researchers is the reassurance to design future studies with taste words, giving more confidence in using online experiments and less pressure to implement real tastants. When attempting to influence actual taste perception, more recent results from Sugimori and Kawasaki’s (2022) study highlighted a qualitative difference in the color-taste correspondence supposedly introduced by the varying intensity of bitterness. Essentially, testing with real tastants could raise and settle questions previously omitted or inaccessible when researchers only experimented with taste words. Consider, for example, how challenging it would be to formulate an experiment that manipulates the intensity of bitterness using only lexical stimuli. Interestingly, the results of these two studies have settled the question of testing with taste words, yet, at the same time, they encourage future studies to depart from the traditional path and consider the gains in adopting naturalistic settings.

## Theoretical Accounts of the Visual-Taste Crossmodal Correspondences

In the previous sections of this review, a few theories attempting to account for the spontaneous associations between taste and a range of features of different visual channels have been mentioned briefly, often as part of the effort to understand the mechanism(s) underpinning crossmodal correspondences. In recent years, progress has undoubtedly been made in our attempts to understand what could have encouraged people to connect taste qualities and specific visual features. Spence and Levitan (2021) evaluated a few popular explanations behind the associations between features of different modalities. Unlike the general consensus concerning the mapping patterns, there remains ongoing debate concerning those factors that may give rise to, or mediate, crossmodal associations. Prominent theories refer to the role of emotion, language, and learning when people infer, or match to color, shape, and taste. However, it appears that none of the currently popular theories can by themselves explain all the variance that has been documented in the data (Spence, 2011; Wang et al., 2016). As Spence and Levitan point out, the various accounts should not be considered as being mutually exclusive when explaining the operation of crossmodal correspondences, each mediating factor might exert a different degree of influence depending on the visual and gustatory stimuli under consideration.

This section provides a summary of the various theories that have been put forward to account for the numerous different visual-taste associations reported to date, as well as evidence that directly or indirectly supports those theories. Where applicable, speculations are made concerning how the various accounts would predict the outcome of popular research questions yet to be addressed, and how novel paradigms can help verify the hypotheses of theoretical questions.

### Internalization of the Multisensory Statistics of the Environment: the Statistical Account

When discussing the origins of color-taste crossmodal correspondences, the formation of these crossmodal associations can be compared to the associative learning that results from accumulated observations, which saw the onset of one sensory feature predicting the likely presence of the other (Spence, 2018). In this regard, the robust association between sweet and red is perhaps based on the adaptive behavior whereby colors are used to determine the ripeness of fruit/leaves (Dominy & Lucas, 2001; Lee et al., 2013; cf. Sumner & Mollon, 2003). In addition to the hue, the saturation of redness has also been shown to correlate with the sugar content present in fleshy fruits such as strawberries (Jia et al., 2013; though people also correlate lower energy levels in foods to a greener appearance, Foroni et al., 2016). It has been suggested that these important statistical correlations in the environment could have contributed to the robust crossmodal correspondence that has been documented between color saturation and taste intensity (Saluja & Stevenson, 2018). Additionally, aeons of fruit foraging (and consumption) activities in human history may have helped to reinforce the expectation of sweetness when seeing redness, or the lack of greenness, in food (Foroni et al., 2016; Lieberman, 2006).

Beyond the associative learning motivated by adaptive behaviors, people may register and internalize the associations between regularly co-occurring stimuli when interacting with, and learning from, concepts and objects in the environment (Barlow, 2001). The projection of these internalized connections to visual-taste inference provides an intuitive explanation for many types of crossmodal correspondence. The statistical account emphasizes the intricate connection between food and its visual appearance, suggesting that people pick-up on color cues when consuming food and internalize them as the association between color and taste (Spence & Levitan, 2021). Such a theory is backed by the evidence that most color-taste associations could be rapidly picked-up by adults (Higgins & Hayes, 2019) and infants (Reardon & Bushnell, 1988). However, for certain associations, the statistical correlations might not be readily available for internalization. For example, the picture is not as clear-cut for shape-taste associations due to the lack of a statistical relationship between geometrical features and taste qualities.

If crossmodal associations are acquired from the environment, there should be changes over years of development in the food and agricultural industries and perhaps some cultural variation too. The shift in the environment can be surprisingly rapid, for instance, linked to the introduction of artificial coloring in food products and increasingly frequent cultural exchange (Kagliwal, 2020). Spence (2021b) highlighted how the connotations of blue food have gradually shifted from it being seen as an artificial color associated with raspberry flavor (e.g., in candy floss and Slush Puppie drinks) to a natural food coloring that is not associated with any particular taste/flavor over the last 70 years or so. However, there does not appear to be a noticeable shift in the general mappings of color-taste correspondences, presumably due to the relatively short recorded history (e.g., Déribéré, 1978). It would certainly be intriguing if the shift over a more extended period could be analyzed retrospectively, such that a comparison could be made before and after the widespread introduction of artificial food colorants (e.g., see Downham & Collins, 2000; Wilson, 2008).

Contextual cues exert a considerable influence over the associations between color and taste (Spence, 2021b); other elements in the environment might be used to make more efficient inferences (Elliot, 2019). It has been suggested that people may draw inferences from external sources to associate taste with color depending on their own idiosyncratic experiences (Schloss et al., 2015), probably involving the weighting of preference, expectation, and context (Meier et al., 2012; Schloss & Palmer, 2017; Spence & Levitan, 2022). However, it remains uncertain how these different sources are weighted in order to calculate the goodness-of-fit of potential matches. It is possible that, when inferring color-taste associations, assessors may not just refer to a specific mediating object but also consider how appropriate their inference will be relating to the other plausible candidates (Schloss et al., 2018).

**Figure 3.**
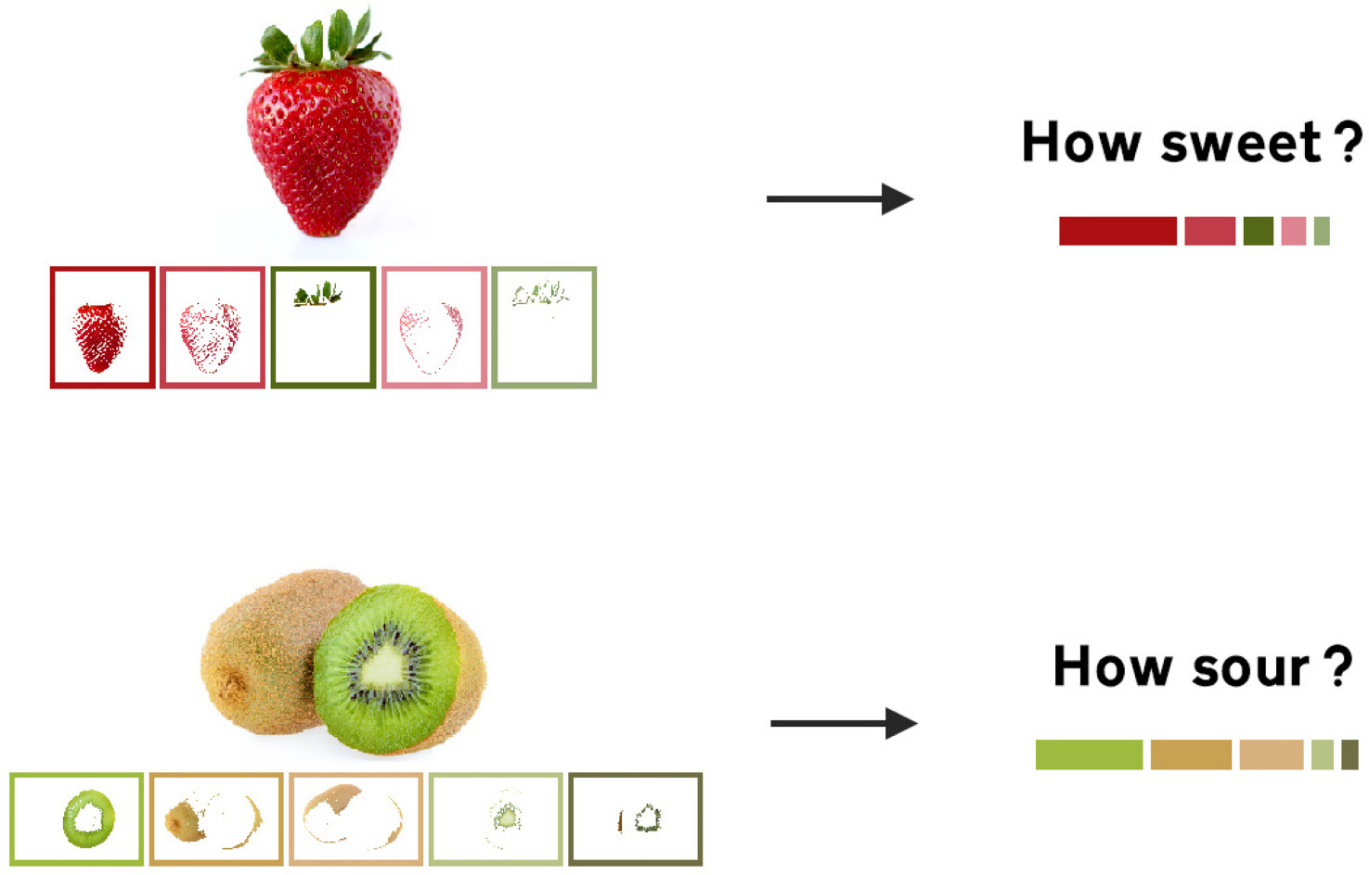
The multicolor paradigm suggested by the current review to examine whether people's access to mediating objects guides their taste expectations.

*Note.* A ribbon is obtained by extracting the typical colors of a fruit image and considering the ratio of each color cluster. The color scheme of this ribbon reflects the appearance of the fruit, which could supposedly remind people of the source object when its taste expectations are being assessed.

Hypothetically, learning how the inference is made from an ensemble of colors could grant researchers an insight into the process underlying color-taste associations. This type of ensemble can be portrayed by a color profile that happens to resemble a ubiquitous mediating source, such as, say, extracting different shades of red and green in a ratio that is matching to the typical image of a strawberry (see Figure 3; with algorithms implemented by Krzywinski, 2022). If people were to estimate the taste of a cluster of colors in this proposed paradigm, their response would likely be regulated by the mediating object more than representing the sum of constituting colors (e.g., Woods et al., 2016). For example, people might find the combination of saturated yellow and black to resemble a ripe banana, therefore associate this color arrangement with sweet instead of sour or bitter. More crucially, the paradigm can be used to manipulate certain information of the context to learn how environmental cues can be used to guide the inference of taste.

#### The Link Between Artificiality and Transparency

Some contextual cues can change how people perceive a visual feature's connotative meaning. For example, artificiality might turn a typically aversive hue (such as blue color in food; see Lee et al., 2013) into a symbol of excitement and attractiveness (see Spence, 2021b). Consider here only how, in everyday life, soft drinks and jellies are the most frequently encountered transparent colored food, which happen to be often artificially dyed in bright colors and constantly associated with a high sugar content (Spence, 2019, 2021b). Supposedly, then, people will associate “seeing through” appearances with artificiality and the presence of sugar. Therefore, it is worth entertaining the idea that color transparency could be used to convey a sense of artificiality in food. However, no known studies have explicitly confirmed this putative relationship between artificiality and transparency.

**Figure 4.**
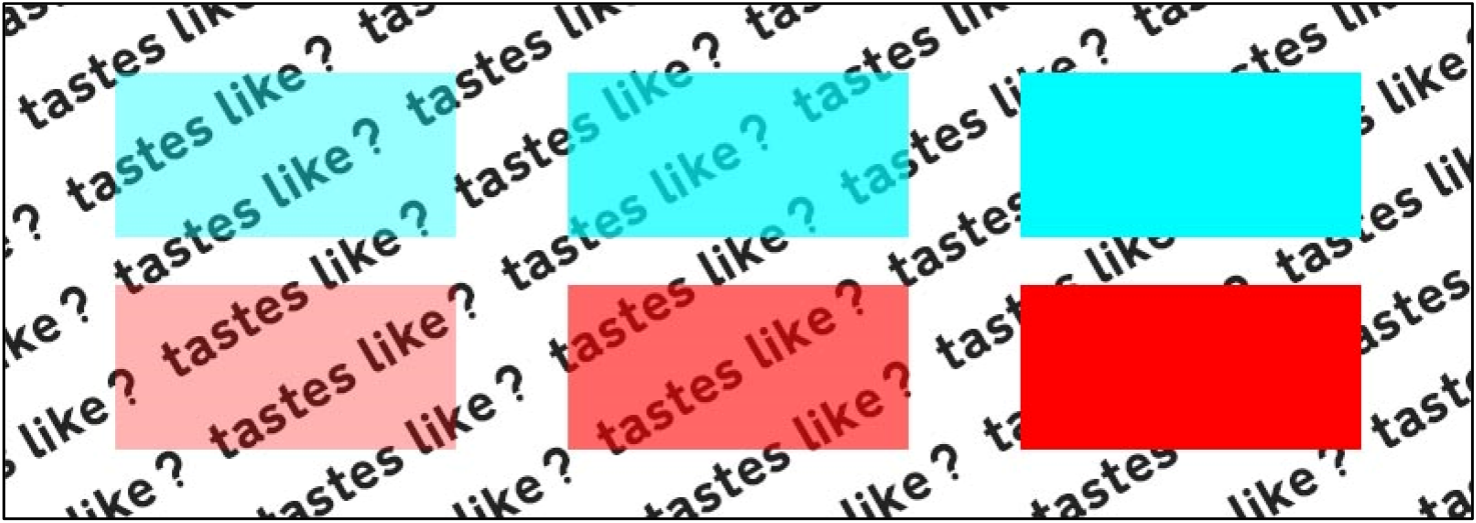
Examples of the visual stimuli varying in their degree of transparency.

**Figure 5.**
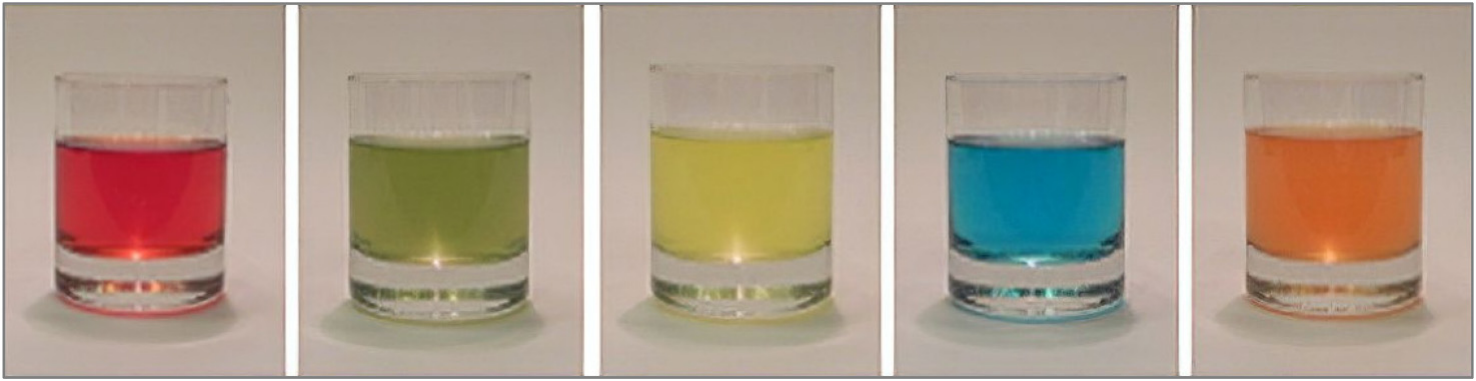
A previous study conducted by Wan et al. (2014a) in which the researchers also presented drinks (beverages) of different colors in a translucent receptacle*.*

*Note*. Adapted from “Does the type of receptacle influence the crossmodal association between color and flavor? A cross-cultural comparison,” by Wan, X., Velasco, C., Michel, C., Mu, B., Woods, A. T., & Spence, C., 2014, *Flavour, 3*(1). CC BY 2.0.

Velasco, Michel et al. (2016) displayed six translucent cups holding the same liquid of different colors and had around 5,000 participants pick the sweetest-looking from among the six colored drinks. Contrary to the conventional color-taste mappings that have been recorded (where blue is seen as the least sweet color; Woods et al., 2016), blue was found to be the second sweetest drink after red. However, it is unknown if the association is mediated by a relevant source object (or drink), such as reminding participants of a specific energy drink or ice slushy. Since Velasco, Michel et al. conducted their experiment in a museum, it is impossible to unravel whether the cup, the liquid, and/or the environment may have provided any contextual clues. Perhaps this can be assessed in a laboratory setting with abstract colors of varying degrees of transparency (see Figure 4), with the see-through effect simulated by creating contrast with background elements. The proposed design could be a simple color-taste matching survey but with transparency being one of the variables, which will allow researchers to investigate the role of “seeing-through” texture (or rather a peculiar visual property). By doing so, the participants will be able to assess the taste of these visual stimuli without being distracted by the product-extrinsic factors such as the connotative properties (or semantic associations) of the receptacle (see Figure 5). Consider the increasing popularity of the blue alcoholic drinks on the supermarket shelf, it might be in the interest of marketers to explore how to put this link between artificiality and transparency to work and control the degree of novelty or excitement their products convey.

#### Mediating Shape-Taste Associations by Means of Crossmodal Statistics

Compared to color, attributes of shape curvilinearity such as roundness and angularity would seem less likely to be internalized along with a given food quality. The appearance of curvilinearity is not as categorical as that of color. It would be easy to label a banana as “yellow” but tricky to definitely categorize the shape of banana (i.e., a dull-edged crescent) as round or angular due to the ambiguity. The discriminability and distinctiveness of color hues would have assisted the generalization of the visual experience as there is little ambiguity among competing cues (Urushihara & Miller; 2009). It is also evident that most natural foods do not possess a predominantly curvy or angular visual appearance; they instead constitute a myriad of geometric features with curvilinearity characteristics that are more or less arbitrary. Although some geometric patterns can occasionally be observed, the internalization was likely prevented by the lack of reliable regularities (Barlow, 2001). Considering the global assignment model used in the inference process as proposed by Schloss et al. (2018) and supported by their study, even if there exist certain food sources appearing with very characteristic curvilinearity features (or even with a basic geometric shape), assessors might reject making the inference since they know that such a connection would not target valid statistical relationship.

The curvilinearity features are not likely to exploit a definite source object as the mediator when being matched with a taste. However, the outlined shapes of an object, composed of various angular and rounded contours, can successfully cue the presence of a mediating object (Okuzumi et al., 2015). Arguably, the connection between the object and its outline is not strictly the product of the internalization of crossmodal statistics, as making these connections does not involve computing the probability of the regularities between modalities. In many cases, it would be the basic executive functions such as object recognition that drives the observer to connect a shape with a particular food (Archibald & Kerns, 1999; Landau et al., 1988). Although not necessarily pertaining to the inference of taste, the food and beverage industry has been enjoying the benefits of object recognition for more than half a century now as, for example, by Jif brand's lemon juice in their iconic lemon-shaped container (Pell, 2021).

**Figure 6.**
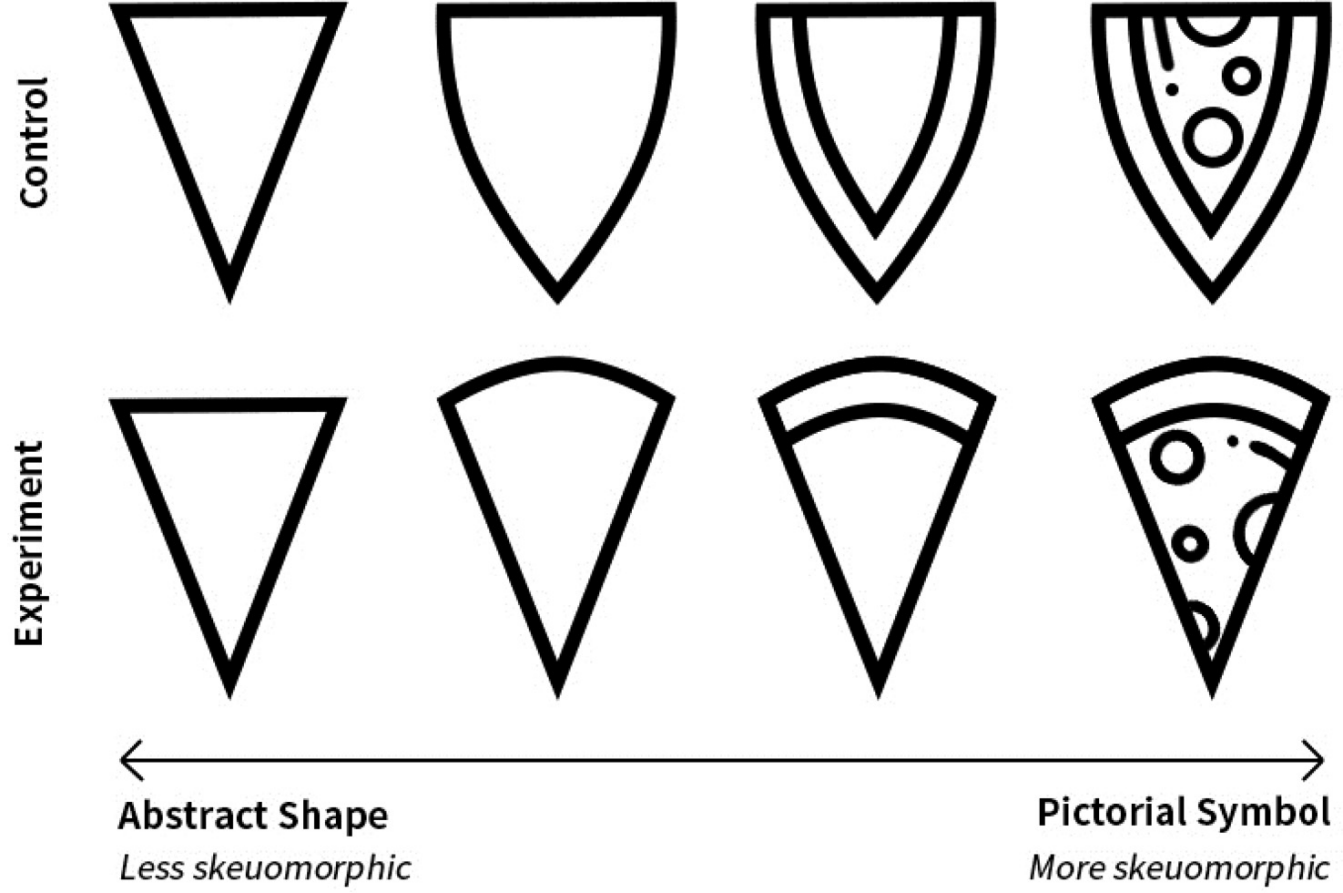
A hypothetical set of stimuli that manipulates the degree of object resemblance to examine the chances of establishing crossmodal correspondences via a mediating object.

*Note*. The bottom set (experimental stimuli) starts to resemble a slice of pizza as additional geometric features are added, while the top set (control stimuli) are designed to be non-specific even with an equal number of geometric features added.

People would presumably associate the silhouette of a lemon with a sour taste (see Demattè et al., 2009) while associating an oval shape of a similar size with a sweet taste. One may wonder how skeuomorphic (i.e., realistic, resembling, or symbolic) a shape must be to convince viewers that they should link the shape to a specific object. A paradigm can be devised to assess the taste association of a spectrum of visual stimuli, ranging from abstract shapes all the way through to pictorial symbols, and determine the degree of realism required for people to start considering the mediating role of a source object (see Figure 6). Assuming that the other mediating factors are rejected whenever a source object is accessible, the participants in this paradigm might suddenly realize (when viewing more and more skeuomorphic shapes on this spectrum) that they are inferring the taste of pizza and immediately associate the stimulus with salty and possibly umami. Alternatively, if different mediating factors cooperate and coexist to help infer the taste of the stimuli, the salty rating will only gradually increase as more geometric features are added to the stimuli to resemble the pictorial symbol of a pizza.

Overall, it would appear that the theory of the internalization of environmental statistics could account for some of the crossmodal correspondences between taste and visual features reasonably well, particularly between taste and color, that have been documented to date. This claim could be further strengthened if a shift in the color-taste mappings can be found and attributed to the changes in the environment over time, such as the widespread application of artificial food dyes (Spence, 2021b) and the development of agricultural products (e.g., selective breeding and domestication of crops; Gracia et al., 2020). On the other hand, because shape features are less likely to generate statistical relationships when appearing with foods, the statistical account is certainly less capable of explaining the taste associations of curvilinearity than of color.

### Crossmodal Correspondences Mediated by Emotions: The Affective Account

Certain crossmodal correspondences are more plausibly mediated by emotions than by reference to the internalized connections of co-occurring stimuli (Wang et al., 2016). As demonstrated by the small number of studies that were reviewed in the previous sections, there has been a trend in investigating the role of valence not only when matching basic taste qualities with visual features, but also in the wider literature on crossmodal correspondences (e.g., Turoman et al., 2018; Velasco et al., 2015b; Velasco et al., 2018b). The *affective account* holds that if different concepts happen to be regarded as possessing a similar hedonic tone, the similarity of the emotion that they induce will inspire the assessors to connect these concepts (Whiteford et al., 2018). For example, most people consider sweetness to be pleasant, and they also happen to like the rounded, curvy shapes supposedly because they find those features approachable (Velasco et al., 2016a; Velasco et al., 2016b; though see Palumbo et al., 2015, for what exactly it is about rounded shapes that makes them preferable). Therefore, according to the affective account, sweet taste and rounded shapes may be associated because both are associated with positive emotion.

When researchers assessed the hedonic values of the concepts being matched in shape-taste correspondences, the associations involving geometric features such as curvilinearity (Velasco et al., 2016b) and symmetry (Salgado-Montejo et al., 2015) would seem to suggest that they are matched because a similar emotion is induced. The basic tastes and their matching geometric features can be closely mapped onto the semantic space of emotion: Sweet, rounded, and symmetric shapes all map to a pleasant emotion, which agrees with the sweet-round and sweet-symmetry associations as reported by the previous studies. Similarly, other taste qualities and geometric features sit closer to the negative emotions when being mapped to the semantic space, which is also in line with patterns observed in other shape-taste associations. The fact that emotion mappings conform with the shape-taste mappings, combined with the lack of statistical correlations for most of the associations between shape and taste, would appear to suggest that the affective account provides a plausible alternative to the statistical account (Spence & Deroy, 2014).

There have also been attempts to assess the hedonic tone of color attributes. So, for example, the hue, saturation, and brightness of color have all been found to manifest a consistent emotional value (Valdez & Mehrabian, 1994). For instance, the color blue and green are liked the most compared to other hues, followed by purple and red, with yellow being the least pleasing color hue (see also Madden et al., 2000). Although the hue category seems to have significantly affected the reported hedonic value of the color stimulus, emotions are not the likely mediator of associations between color and taste. The trend of blue and green being rated as the most pleasant hues does not appear to agree with the mappings of color-taste associations (see Table 1), as otherwise, they should be regarded as the best candidates for the sweet taste to match with. The mediation of crossmodal correspondences likely involves a certain degree of competition between different factors. Judging from the evidence presented thus far, internalized regularities, especially concerning a specific object, are the most effective mediation route for visual-taste associations when a meaningful statistical correlation is available (Spence & Levitan, 2022).

Meanwhile, researchers have also investigated the hedonic tone of various gustatory properties (such as quality and intensity) independent of visual features. The relationship between gustatory experience and emotion is not defined solely by the taste quality but also by the intensity of taste perception (Herbert et al., 2014). A recent study by Wu and Jiang (2022) reported that valence and taste intensity were correlated in a two-step relationship: There appears to be a literal “sweet spot” for the concentration of sucrose solution; only when the concentration level is within the proximity of this concentration do participants like the stimulus more as the taste gets sweeter (cf. Kim et al., 2014; Yang et al., 2019). Hypothetically, should this effect translate to visual-taste crossmodal correspondences, there could potentially be a range of concentrations where the taste stimuli might match more consistently with positively-valenced design features such as round shape and the color blue. That being said, associations between taste and color, similar to those between taste and music pitch, are less likely to be affected by emotions (Crisinel & Spence, 2012).

Between the statistical and affective accounts, the evidence presented thus far would appear to suggest that certain types of crossmodal correspondences are better explained by one account rather than the others. However, not much is known about what might have driven one mediator to prevail over another. In fact, there is little knowledge concerning whether the underlying mechanism is a competition or cooperation between the potential mediators. Worse still, the route of mediation cannot be determined by the modality that happens to be assessed. The taste-color correspondences are likely mediated by statistics while the taste-shape correspondences are likely mediated by emotion. Likewise, the crossmodal correspondences involving color would appear to have exploited internalized statistics of the environment when matched with taste (Velasco, Michel et al., 2016), but are better explained by the affective account when matched with fine fragrances (Schifferstein & Tanudjaja, 2004). When comparing the two accounts, there would seem to be a tendency to base decisions on internalized statistics should they be readily available / accessible. A novel paradigm such as the “spectrum of resemblance” proposed above (see Figure 6) might provide an appropriate approach to examining the interaction of mediating factors.

#### Processing Fluency

The term *processing fluency* describes the positive effect associated with processing easy and stress-free stimuli (or stimulus combinations) (Reber, 2011). Some researchers have also expanded this idea to cover the typicality effect, citing that people also like seeing what they expect (Vogel et al., 2021). Theoretically, presenting features from different sensory modalities that happen to agree (associate) with each other (either via mediation of emotion or taste correspondences) can give rise to enhanced processing fluency (Winkielman et al., 2015) as when dining experiences are enhanced by dishes that confirm, or are congruent, with customers’ expectations (e.g., Spence et al., 2015). The rationale here is to create a visual stimulus that is fluent and familiar so that it will create a positive hedonic tone when it is processed (Reber et al., 1998). This, in turn, should lead the stimulus to be more likely associated with sweet taste (or less associated with sour taste).

In a dining environment, the appraisal of certain combinations of color and shape has shown signs that such arrangements are potentially being processed fluently. Stewart and Goss (2013) found the shape and color of the plate to have a significant interaction effect on the liking and perceived sweetness of the food on the plate. Curiously, their results showed that food on the black and square plate received a similar rating (if not higher in some cases) of hedonic value than the same food served on the white and round plate. Here, the surprise comes from the fact that when black color and square shape were assessed independently, they were less liked than white and round. Also, when presented as an abstract concept without context or accompanying features, the black color and square shape are typically considered the “bitter color” or the “bitter shape.” Stewart and Goss's findings give an example of processing fluency found in graphical features with similar emotional value. Perhaps even more interestingly, only a limited portion of this emotion was translated to the rating of sweetness. Relevant to the proposed competition between different theories accounting for visual-taste correspondences, this could be the evidence that some visual information (likely color in this case) is less prone to the mediation of emotion.

While on the topic of visual fluency, the century-old idea of the Kandinsky correspondences has been suggesting the universal associations between color and shape, based on the belief that visual features are fundamentally interconnected (see Dreksler & Spence, 2019). Supposedly, if people were asked to infer the taste of an object with a color and shape conforming to one of the Kandinsky correspondences, the perceptual fluency and the valence it induces would make the estimated taste sweeter. The problem, though, is that the color-shape correspondences examined by researchers thus far have not revealed a consistent pattern. The validity and reliability of the systematic mappings between color and shape are still debated in the literature (Chen et al., 2015; Song et al., 2022). Despite being among the most investigated visual features, there is not even a general conclusion on the associations between color and curvilinearity (Malfatti, 2014). At this point, it would seem that further empirical evidence is needed before designers can confidently exploit the fluency of shape and color to enhance consumers’ experience.

It is also worth considering how typicality could induce a positive emotion through familiarity or by meeting expectations (Vogel et al., 2021). A product with sensory features that conform to the anticipation of its assessors will generally give rise to a positively-valenced experience (Sundar & Noseworthy, 2016). Relevant to the crossmodal correspondences between color and taste, the color cues were reported to suffer from a diminished effect on taste perception when the actual gustatory experience diverges too much from the expectation (Sugimori & Kawasaki, 2022), though this theory has not been fully verified (Wang et al., 2017). It should also be noted that in this case, instead of a modified valence by the incongruent elements, it is more likely that the drastic contrast between expectation and actual perception has completely dissuaded the assessors from relying on visual cues (Carvalho & Spence, 2019).

In summary, a wealth of literature on the crossmodal correspondences has attempted to identify the underlying operations that have led to the associations between taste qualities and different visual features (Deroy & Spence, 2016). By now, the researchers have narrowed the search to a few promising theories. For the color-taste and shape-taste associations that have been assessed in this review, the evidence and former investigations suggested them to be driven by two particular accounts: Statistical and affective; the former attributes the connections to the spontaneous internalization of the statistics of the environment, while the latter stresses the mediating role of emotion. According to a series of supplementary studies, it would seem that the two explanations are also subject to being mediated by other factors, such as the reinforcement of the adaptive memory (environmental information that is relevant to survival; Nairne et al., 2007) and the processing fluency that modulates emotional appraisal (Spence et al., 2015; Sundar & Noseworthy, 2016). While the evidence is scarce, a hierarchy (or priority) system is likely responsible for determining how assessors arrive at their inference. For instance, although both color and shape possess certain valence values, people seem to have disregarded the affective quality of color when making color-taste associations. When comparing the ways in which visual features are associated with taste, there seems to be a fair possibility that the affective account functions as a backup option for establishing crossmodal correspondences when mediation via multisensory object representations is unavailable. The current stage of theoretical understanding remains insufficient to determine how different routes of mediation would operate together, but the interaction of those factors involved could be further assessed with specific paradigms.

While the research interest has primarily focused on the two discussed theories as they hold the best prospect of explaining crossmodal associations, there is no reason to exclude the mediation of other factors. Acknowledging the potential of these factors could lead to the discovery of new models that may help supplement the current ones. Relevant here, there is likely a sizable reward if future interests can be directed to consider other factors in the vast semantic space (Osgood et al., 1957/1967, pp. 34–39). Given how the current explanations do not appear to catch all the variance in the mappings (e.g., Velasco et al., 2016a), there might be a surprisingly good chance to discover factorial clusters (defined as *dimensions of meaning* by Osgood et al.) that are better able of describing and conceptualizing the visual stimuli (e.g., Weierich et al., 2010; Wendt, 1968). This could be exploring other dimensions of meaning apart from the valence value (largely captured by the *evaluation* dimension; represented by the “good–bad” scale), such as *activity* (“active–passive” scale) and *potency* (“strong–weak” scale). For example, the effect of tastant concentration on shape-taste correspondences can be confirmed by comparing the potency (taste intensity) and evaluation (emotion) factors in semantic differential analyses. Research has also revealed other dimensions that could be relevant when examining other potential mediators, such as the cluster of expectation, excitement, and typicality (relevant to the dimension of *familiarity*; Bentler & LaVoie, 1972) or the effect of fluency in terms of cognitive load (relevant to the dimension of *complexity*; Petrenko; 1993, as cited in Trofimova, 1999). It is also possible, albeit a remote possibility, that other of the chemical senses (e.g., olfaction) could have been mediating the taste correspondences via crossmodal similarity (e.g., Halabi & Saleh, 2021).

## Conclusions

After decades of research effort over the past decades, the literature documenting the crossmodal correspondences between visual features and taste has accumulated a comprehensive understanding of these associations. Researchers have long arrived at the consensus that color hue and a handful of geometric features are consistently matched with the basic taste qualities. In recent decades, it has also been shown that the pattern of their associations can be used to create specific visual designs that manipulate the appraisal and perception of taste under certain circumstances. With these fundamental understandings established, the subsequent studies grew diverse and touched on various research questions ranging from cultural validity to real-life applications. Overall, it is reassuring to observe a global consistency of the matching patterns across different cultures, although some weaker connections have also been identified. Notably, the association between shape curvilinearity and taste qualities has not always appeared to be as robust as that recorded between color hue and taste.

When comparing the popular theoretical models accounting for the crossmodal correspondences, it is intriguing to note how the mediating process, or the causes leading people to draw taste matches, can be remarkably different for color and geometric features. It would seem that an intuitive mediating route exists for color-taste associations, which can be attributed to the internalization of the crossmodal statistics of the environment. However, when a mediating object is unavailable, such as when matching geometric features with taste, people appear to match unrelated stimuli on the basis of the emotion that is associated with the component perceptual stimuli. Unfortunately, there has been little research concerning the interaction of these explanations, which would require novel paradigms to examine the competition of different mediating factors. It is believed that further studies should consider mapping the relevant perceptual concepts to a semantic space, which will allow the crossmodal correspondences to be quantified and compared on meaningful scales of emotion, potency, and activity. Doing so will also provide a fair chance to reveal other factors responsible for mediating the associations.

## References

[bibr1-20416695221127325] Adler E. Hoon M. A. Mueller K. L. Chandrashekar J. Ryba N. J. P. Zuker C. S. (2000). A novel family of mammalian taste receptors. Cell, 100(6), 693–702. 10.1016/S0092-8674(00)80705-9 10761934

[bibr2-20416695221127325] Aoki K. Akai K. (2022). A comparison between Spain and Japan with respect to the color, expected taste scale, and sustainability effects of strawberries: A choice experiment. Food Quality and Preference, (103), Article 104671. 10.1016/j.foodqual.2022.104671

[bibr3-20416695221127325] Archibald S. J. Kerns K. A. (1999). Identification and description of new tests of executive functioning in children. Child Neuropsychology, 5(2), 115–129. 10.1076/chin.5.2.115.3167

[bibr4-20416695221127325] Barlow H. (2001). The exploitation of regularities in the environment by the brain. Behavioral and Brain Sciences, 24(4), 602–607. 10.1017/S0140525X01000024 12048943

[bibr5-20416695221127325] Bentler P. M. LaVoie A. L. (1972). An extension of semantic space. Journal of Verbal Learning and Verbal Behavior, 11(2), 174–182. 10.1016/S0022-5371(72)80074-4

[bibr6-20416695221127325] Brillat-Savarin J. A. (1835). Physiologie du goût [The philosopher in the kitchen / The physiology of taste]. J. P. Meline: Bruxelles. Translated by A. Lalauze (1884), A handbook of gastronomy. Nimmo & Bain.

[bibr7-20416695221127325] Carleton A. Accolla R. Simon S. A. (2010). Coding in the mammalian gustatory system. Trends in Neurosciences, 33(7), 326–334. https://doi.org/dv5q52 20493563 10.1016/j.tins.2010.04.002 PMC2902637

[bibr8-20416695221127325] Carvalho F. M. Spence C. (2019). Cup colour influences consumers’ expectations and experience on tasting specialty coffee. Food Quality and Preference, 75, 157–169. 10.1016/j.foodqual.2019.03.001

[bibr9-20416695221127325] Cecchini M. P. Knaapila A. Hoffmann E. Boschi F. Hummel T. Iannilli E. (2019). A cross-cultural survey of umami familiarity in European countries. Food Quality and Preference, 74, 172–178. 10.1016/j.foodqual.2019.01.017

[bibr10-20416695221127325] Chen N. Tanaka K. Watanabe K. (2015). Color-shape associations revealed with implicit association tests. PLOS ONE, 10(1), Article e0116954. 10.1371/journal.pone.0116954 25625717 PMC4308101

[bibr11-20416695221127325] Chen Y.-C., Huang P.-C., Woods A., & Spence C. (2016). When “Bouba” equals “Kiki”: Cultural commonalities and cultural differences in sound-shape correspondences. Scientific Reports, 6(1), Article 26681. https://doi.org/gfrjdg 10.1038/srep26681 PMC4882484 27230754

[bibr12-20416695221127325] Cheskin L. (1967). Secrets of marketing success: An expert’s view on the science and art of persuasive selling. Simon and Schuster.

[bibr13-20416695221127325] Chitturi R. Londono J. Amezquita C. (2019). The influence of color and shape of package design on consumer preference: The case of orange juice. International Journal of Innovation and Economic Development, 5(2), 42–56. 10.18775/ijied.1849-7551-7020.2015.52.2003

[bibr14-20416695221127325] Crisinel A.-S. Spence C. (2012). The impact of pleasantness ratings on crossmodal associations between food samples and musical notes. Food Quality and Preference, 24(1), 136–140. 10.1016/j.foodqual.2011.10.007

[bibr15-20416695221127325] Cuskley C. Dingemanse M. Kirby S. van Leeuwen T. M. (2019). Cross-modal associations and synesthesia: Categorical perception and structure in vowel-color mappings in a large online sample. Behavior Research Methods, 51(4), 1651–1675. 10.3758/s13428-019-01203-7 30945162 PMC6691033

[bibr16-20416695221127325] Cytowic R. E. Wood F. B. (1982). Synesthesia. II. Psychophysical relations in the synesthesia of geometrically shaped taste and colored hearing. Brain and Cognition, 1(1), 36–49. 10.1016/0278-2626(82)90005-7 6927553

[bibr17-20416695221127325] Demattè M. L. Sanabria D. Spence C. (2009). Olfactory discrimination: When vision matters? Chemical Senses, 34(2), 103–109. https://doi.org/dnngk2 18794200 10.1093/chemse/bjn055

[bibr18-20416695221127325] Déribéré M. (1978). Relationship between perfumes and colors. Color Research & Application, 3(3), 115–116. 10.1002/col.5080030307

[bibr19-20416695221127325] Deroy O. Spence C. (2016). Crossmodal correspondences: Four challenges. Multisensory Research, 29(1–3), 29–48. 10.1163/22134808-00002488 27311290

[bibr20-20416695221127325] Deroy O. Valentin D. (2011). Tasting liquid shapes: Investigating the sensory basis of cross-modal correspondences. Chemosensory Perception, 4(3), 80–90. 10.1007/s12078-011-9097-1

[bibr21-20416695221127325] Desimone R. Schein S. J. Moran J. Ungerleider L. G. (1985). Contour, color and shape analysis beyond the striate cortex. Vision Research, 25(3), 441–452. 10.1016/0042-6989(85)90069-0 4024463

[bibr22-20416695221127325] Dichter E. (1971). The strategy of selling with packaging. Package Engineering Magazine, 7, 16a–16c. Hagley Museum and Library Archives. https://findingaids.hagley.org/repositories/3/archival_objects/228752

[bibr23-20416695221127325] Dominy N. J. Lucas P. W. (2001). Ecological importance of trichromatic vision to primates. Nature, 410(6826), 363–366. 10.1038/35066567 11268211

[bibr24-20416695221127325] Downham A. Collins P. (2000). Colouring our foods in the last and next millennium. International Journal of Food Science & Technology, 35(1), 5–22. 10.1046/j.1365-2621.2000.00373.x

[bibr25-20416695221127325] Dreksler N. Spence C. (2019). A critical analysis of colour-shape correspondences: Examining the replicability of colour-shape associations. i-Perception, 10(2), 1–34. 10.1177/2041669519834042 PMC6442080 30956786

[bibr26-20416695221127325] Duffy V. B. Hayes J. E. Bartoshuk L. M. Snyder D. J. (2009). Taste: Vertebrate psychophysics. In Squire L. R (Ed.), Encyclopedia of neuroscience (pp. 881–886). Elsevier. 10.1016/B978-008045046-9.01673-9

[bibr27-20416695221127325] Elliot A. J. (2019). A historically based review of empirical work on color and psychological functioning: Content, methods, and recommendations for future research. Review of General Psychology, 23(2), 177–200. 10.1037/gpr0000170

[bibr28-20416695221127325] Ernst S. B. (1980). Color and und et communication. Favre, Jean-Paul and Andre November. Zurich: ABC Verlag. 1979. 167 pages [review of the book *Color und communication*, by J. Favre & A. November]. Journal of Advertising, 9(1), 45. 10.1080/00913367.1980.10673308

[bibr29-20416695221127325] Favre J. November A. (1979). Color und communication [Colour and communication]. ABC Verlag.

[bibr30-20416695221127325] Felleman D. J. Rakic P. (1991). Distributed hierarchical processing in the primate cerebral cortex. Cerebral Cortex, 1(1), 1–47. https://doi.org/db7c5v 1822724 10.1093/cercor/1.1.1-a

[bibr31-20416695221127325] Foroni F., Pergola G., & Rumiati R. I. (2016). Food color is in the eye of the beholder: The role of human trichromatic vision in food evaluation. Scientific Reports, 6(1), Article 37034. 10.1038/srep37034 PMC5107980 27841327

[bibr32-20416695221127325] Goodale M. A. Meenan J. P. Bülthoff H. H. Nicolle D. A. Murphy K. J. Racicot C. I. (1994). Separate neural pathways for the visual analysis of object shape in perception and prehension. Current Biology, 4(7), 604–610. 10.1016/S0960-9822(00)00132-9 7953534

[bibr33-20416695221127325] Gracia A. Sánchez A. M. Jurado F. Mallor C. (2020). Making use of sustainable local plant genetic resources: Would consumers support the recovery of a traditional purple carrot? Sustainability, 12(16), 6549. 10.3390/su12166549

[bibr34-20416695221127325] Halabi O. Saleh M. (2021). Augmented reality flavor: Cross-modal mapping across gustation, olfaction, and vision. Multimedia Tools and Applications, 80(30), 36423–36441. 10.1007/s11042-021-11321-0 34512111 PMC8419827

[bibr35-20416695221127325] Henrich J. Heine S. J. Norenzayan A. (2010). The weirdest people in the world? Behavioral and Brain Sciences, 33(2–3), 61–83. https://doi.org/c9j35b 20550733 10.1017/S0140525X0999152X

[bibr36-20416695221127325] Herbert C. Platte P. Wiemer J. Macht M. Blumenthal T. D. (2014). Supertaster, super reactive: Oral sensitivity for bitter taste modulates emotional approach and avoidance behavior in the affective startle paradigm. Physiology & Behavior, 135, 198–207. 10.1016/j.physbeh.2014.06.002 24912136

[bibr37-20416695221127325] Higgins M. J. Hayes J. E. (2019). Learned color taste associations in a repeated brief exposure paradigm. Food Quality and Preference, 71, 354–365. https://doi.org/ggbjft

[bibr38-20416695221127325] Hutmacher F. (2019). Why is there so much more research on vision than on any other sensory modality? Frontiers in Psychology, 10, Article 2246. https://doi.org/gg6sh2 10.3389/fpsyg.2019.02246 PMC6787282 31636589

[bibr39-20416695221127325] Ikeda K. (1909/2002). New seasonings. Chemical Senses, 27(9), 847–849. 10.1093/chemse/27.9.847 12438213

[bibr40-20416695221127325] Jacquot M. Noel F. Velasco C. Spence C. (2016). On the colours of odours. Chemosensory Perception, 9(2), 79–93. https://doi.org/f8tp9j

[bibr41-20416695221127325] Jia H. Wang Y. Sun M. Li B. Han Y. Zhao Y. Li X. Ding N. Li C. Ji W. Jia W. (2013). Sucrose functions as a signal involved in the regulation of strawberry fruit development and ripening. New Phytologist, 198(2), 453–465. 10.1111/nph.12176 23425297

[bibr42-20416695221127325] Kagliwal B. (2020). Visualising taste: How business changed the look of what you eat by Ai Hisano (review) [review of the book *Visualizing taste: How business changed the look of what you eat*, by A. Hisano]. Technology and Culture, 61(4), 1224–1226. https://bit.ly/3xx1I1O

[bibr43-20416695221127325] Kalat J. W. Rozin P. (1973). “Learned safety” as a mechanism in long-delay taste-aversion learning in rats. Journal of Comparative and Physiological Psychology, 83(2), 198–207. 10.1037/h0034424 4706590

[bibr44-20416695221127325] Kim J.-Y. Prescott J. Kim K.-O. (2014). Patterns of sweet liking in sucrose solutions and beverages. Food Quality and Preference, 36, 96–103. 10.1016/j.foodqual.2014.03.009

[bibr45-20416695221127325] Koch C. Koch E. C. (2003). Preconceptions of taste based on color. The Journal of Psychology, 137(3), 233–242. 10.1080/00223980309600611 12795546

[bibr46-20416695221127325] Krzywinski M. (2022). Image color summarizer (Version 0.67) [Web-based computer software]. http://mkweb.bcgsc.ca/color-summarizer

[bibr47-20416695221127325] Kurihara K. (2015). Umami the fifth basic taste: History of studies on receptor mechanisms and role as a food flavor. BioMed Research International, 2015, Article 189402. 10.1155/2015/189402 PMC4515277 26247011

[bibr48-20416695221127325] Landau B. Smith L. B. Jones S. S. (1988). The importance of shape in early lexical learning. Cognitive Development, 3(3), 299–321. 10.1016/0885-2014(88)90014-7

[bibr49-20416695221127325] Lee S. M. Lee K. T. Lee S. H. Song J. K. (2013). Origin of human colour preference for food. Journal of Food Engineering, 119(3), 508–515. 10.1016/j.jfoodeng.2013.06.021

[bibr50-20416695221127325] Liang P. Biswas P. Vinnakota S. Fu L. Chen M. Quan Y. Zhan Y. Zhang G. Roy S. (2016). Invariant effect of vision on taste across two Asian cultures: India and China. Journal of Sensory Studies, 31(5), 416–422. 10.1111/joss.12225

[bibr51-20416695221127325] Lieberman L. S. (2006). Evolutionary and anthropological perspectives on optimal foraging in obesogenic environments. Appetite, 47(1), 3–9. 10.1016/j.appet.2006.02.011 16806580

[bibr52-20416695221127325] Madden T. J. Hewett K. Roth M. S. (2000). Managing images in different cultures: A cross-national study of color meanings and preferences. Journal of International Marketing, 8(4), 90–107. https://doi.org/fk5f4b

[bibr53-20416695221127325] Makin A. D. J. Pecchinenda A. Bertamini M. (2012). Implicit affective evaluation of visual symmetry. Emotion, 12(5), 1021–1030. 10.1037/a0026924 22251051

[bibr54-20416695221127325] Malfatti M. (2014). Shape-to-color associations in non-synesthetes: Perceptual, emotional, and cognitive aspects [Doctoral dissertation, University of Trento]. Unitn-eprints PhD. https://bit.ly/3litcm7

[bibr55-20416695221127325] Marks L. E. (1978). The unity of the senses: Interrelations among the modalities. Academic Press. https://bit.ly/394MRDB

[bibr56-20416695221127325] Marks L. E. (1996). On perceptual metaphors. Metaphor and Symbol, 11(1), 39–66. 10.1207/s15327868ms1101_3

[bibr57-20416695221127325] McLaughlin S. Margolskee R. F. (1994). The sense of taste. American Scientist, 82(6), 538–545. Retrieved from https://www.jstor.org/stable/29775325

[bibr58-20416695221127325] Meier B. P. D’Agostino P. R. Elliot A. J. Maier M. A. Wilkowski B. M. (2012). Color in context: Psychological context moderates the influence of red on approach- and avoidance-motivated behavior. PLOS ONE, 7(7), Article e40333. 10.1371/journal.pone.0040333 PMC3394796 22808136

[bibr59-20416695221127325] Mick G. D. (1986). Consumer research and semiotics: Exploring the morphology of signs, symbols, and significance. Journal of Consumer Research, 13(2), 196–213. https://www.jstor.org/stable/2489226

[bibr60-20416695221127325] Mura E. Taruno A. Yagi M. Yokota K. Hayashi Y. (2018). Innate and acquired tolerance to bitter stimuli in mice. PLOS ONE, 13(12), Article e0210032. 10.1371/journal.pone.0210032 PMC6312290 30596779

[bibr61-20416695221127325] Nairne J. S. Thompson S. R. Pandeirada J. N. S. (2007). Adaptive memory: Survival processing enhances retention. Journal of Experimental Psychology: Learning, Memory, and Cognition, 33(2), 263–273. https://doi.org/cp6z9w 17352610 10.1037/0278-7393.33.2.263

[bibr62-20416695221127325] Naor-Raz G. Tarr M. J. Kersten D. (2003). Is color an intrinsic property of object representation? Perception, 32(6), 667–680. 10.1068/p5050 12892428

[bibr63-20416695221127325] Nelson M. R. Hitchon J. C. (1999). Loud tastes, colored fragrances, and scented sounds: How and when to mix the senses in persuasive communications. Journalism & Mass Communication Quarterly, 76(2), 354–372. https://journals.sagepub.com/doi/10.1177/107769909907600212

[bibr64-20416695221127325] Ngo M. K. Misra R. Spence C. (2011). Assessing the shapes and speech sounds that people associate with chocolate samples varying in cocoa content. Food Quality and Preference, 22(6), 567–572. 10.1016/j.foodqual.2011.03.009

[bibr65-20416695221127325] Okuzumi H. Ikeda Y. Otsuka N. Saito R. Oi Y. Hirata S. Haishi K. Kokubun M. (2015). Stroop-like interference in the fruit Stroop test in typical development. Psychology, 6(5), 643–649. https://doi.org/hnjz

[bibr66-20416695221127325] O’Mahony M. (1983). Gustatory responses to nongustatory stimuli. Perception, 12(5), 627–633. 10.1068/p120627 6676714

[bibr67-20416695221127325] O’Mahony M. Goldenberg M. Stedmon J. Alford J. (1979). Confusion in the use of the taste adjectives ‘sour’ and ‘bitter.’ Chemical Senses, 4(4), 301–318. 10.1093/chemse/4.4.301

[bibr68-20416695221127325] Osgood C. E. Suci G. J. Tannenbaum P. H. (1957/1967). The measurement of meaning. University of Illinois Press. https://bit.ly/3sAEjdW (Original work published 1957)

[bibr69-20416695221127325] Palumbo L. Ruta N. Bertamini M. (2015). Comparing angular and curved shapes in terms of implicit associations and approach/avoidance responses. PLOS ONE, 10(10), Article e0140043. 10.1371/journal.pone.0140043 PMC4603793 26460610

[bibr70-20416695221127325] Pedović I. Stosić M. (2018). A comparison of verbal and sensory presentation methods in measuring crossmodal correspondence within a semantic-based approach. Ceskoslovenska Psychologie, 62(6), 602–615. http://cspsych.psu.cas.cz/result.php?id=1043

[bibr71-20416695221127325] Pell K. (2021, June 2). *Jif lemon bottle* [Object]. Museum of Design in Plastics, Arts University Bournemouth, Dorset, UK. https://bit.ly/3yHQE3M

[bibr72-20416695221127325] Petrenko V. (1993). Meaning as a unit of consciousness. Journal of Russian & East European Psychology, 31(2), 5–19. 10.2753/RPO1061-040531025

[bibr73-20416695221127325] Pickford R. W. (1948). Colour blindness in the left eye following an accident. British Journal of Psychology, 39(2), 73–83. 10.1111/j.2044-8295.1948.tb00206.x 18111546

[bibr74-20416695221127325] Piqueras-Fiszman B. Alcaide J. Roura E. Spence C. (2012). Is it the plate or is it the food? Assessing the influence of the color (black or white) and shape of the plate on the perception of the food placed on it. Food Quality and Preference, 24(1), 205–208. 10.1016/j.foodqual.2011.08.011

[bibr75-20416695221127325] Raevskiy A. Bubnov I. Chen Y.-C. Sakai N. (2022). Differences in color representations of tastes: Cross-cultural study among Japanese, Russian and Taiwanese. In P. L. P. Rau (Eds.), HCII 2022 Lecture notes in computer science: Vol. 13311. Cross-cultural design. interaction design across cultures. (pp. 378–395). Springer. 10.1007/978-3-031-06038-0_28

[bibr76-20416695221127325] Razumiejczyk E. Macbeth G. Marmolejo-Ramos F. Noguchi K. (2015). Crossmodal integration between visual linguistic information and flavour perception. Appetite, 91, 76–82. 10.1016/j.appet.2015.03.035 25843936

[bibr77-20416695221127325] Reardon P. Bushnell E. W. (1988). Infants’ sensitivity to arbitrary pairings of color and taste. Infant Behavior and Development, 11(2), 245–250. 10.1016/S0163-6383(88)80010-9

[bibr78-20416695221127325] Reber R. (2011). Processing fluency, aesthetic pleasure, and culturally shared taste. In Shimamura A. P. Palmer S. E. (Eds.), Aesthetic science connecting minds, brains, and experience (pp. 223–242). Oxford University Press. 10.1093/acprof:oso/9780199732142.003.0055

[bibr79-20416695221127325] Reber R. Winkielman P. Schwarz N. (1998). Effects of perceptual fluency on affective judgments. Psychological Science, 9(1), 45–48. 10.1111/1467-9280.00008

[bibr80-20416695221127325] Rolschau K. Wang J. Q. Otterbring T. (2020). Seeing sweet and choosing sour: Compensatory effects of typeface on consumers’ choice behavior. Food Quality and Preference, 85, Article 103964. https://doi.org/hqg2

[bibr81-20416695221127325] Salgado-Montejo A. Alvarado J. A. Velasco C. Salgado C. J. Hasse K. Spence C. (2015). The sweetest thing: The influence of angularity, symmetry, and the number of elements on shape-valence and shape-taste matches. Frontiers in Psychology, 6, Article 1382. 10.3389/fpsyg.2015.01382 PMC4569812 26441757

[bibr82-20416695221127325] Saluja S. Stevenson R. J. (2018). Cross-modal associations between real tastes and colors. Chemical Senses, 43(7), 475–480. https://doi.org/gdnvv8 29868904 10.1093/chemse/bjy033

[bibr83-20416695221127325] Schifferstein H. N. J. Tanudjaja I. (2004). Visualising fragrances through colours: The mediating role of emotions. Perception, 33(10), 1249–1266. 10.1068/p5132 15693669

[bibr84-20416695221127325] Schloss K. B. Hawthorne-Madell D. Palmer S. E. (2015). Ecological influences on individual differences in color preference. Attention, Perception, & Psychophysics, 77(8), 2803–2816. 10.3758/s13414-015-0954-x 26272366

[bibr85-20416695221127325] Schloss K. B. Lessard L. Walmsley C. S. Foley K. (2018). Color inference in visual communication: The meaning of colors in recycling. Cognitive Research: Principles and Implications, 3(1), Article 5. 10.1186/s41235-018-0090-y PMC5820393 29497689

[bibr86-20416695221127325] Schloss K. B. Palmer S. E. (2017). An ecological framework for temporal and individual differences in color preferences. Vision Research, 141, 95–108. 10.1016/j.visres.2017.01.010 28456532

[bibr87-20416695221127325] Simner J. Cuskley C. Kirby S. (2010). What sound does that taste? Cross-modal mappings across gustation and audition. Perception, 39(4), 553–569. 10.1068/p6591 20515002

[bibr88-20416695221127325] Simner J. Ward J. Lanz M. Jansari A. Noonan K. Glover L. Oakley D. A. (2005). Non-random associations of graphemes to colours in synaesthetic and non-synaesthetic populations. Cognitive Neuropsychology, 22(8), 1069–1085. 10.1080/02643290500200122 21038290

[bibr89-20416695221127325] Song J. Shin H. Park M. Nam S. Kim C.-Y. (2022). Complex shapes are bluish, darker, and more saturated; Shape-color correspondence in 3D object perception. Frontiers in Psychology, 13, Article 854574. 10.3389/fpsyg.2022.854574 PMC9114860 35602700

[bibr90-20416695221127325] Spence C. (2011). Crossmodal correspondences: A tutorial review. Attention, Perception, & Psychophysics, 73(4), 971–995. 10.3758/s13414-010-0073-7 21264748

[bibr91-20416695221127325] Spence C. (2012). Managing sensory expectations concerning products and brands: Capitalising on the potential of sound and shape symbolism. Journal of Consumer Psychology, 22(1), 37–54. https://doi.org/ddqbqb

[bibr492-20416695221127325] Spence, C. (2018, October 20-22). *Crossmodal correspondences: Looking for links between sound symbolism & synaesthesia, & their application to multisensory marketing* [Seminar]. Seminar on psychology and brain sciences related to hearing and multisensory processing, Sendai, Japan. https://bit.ly/3Mqfsl7

[bibr92-20416695221127325] Spence C. (2019). On the changing colour of food & drink. International Journal of Gastronomy and Food Science, 17, Article 100161. https://doi.org/gnkqcr

[bibr93-20416695221127325] Spence C. (2021a). The multisensory design of pharmaceuticals and their packaging. Food Quality and Preference, 91, Article 104200. https://doi.org/hgtz

[bibr94-20416695221127325] Spence C. (2021b). What’s the story with blue steak? On the unexpected popularity of blue foods. Frontiers in Psychology, 12, Article 638703. https://doi.org/hn65 10.3389/fpsyg.2021.638703 PMC7960775 33737898

[bibr95-20416695221127325] Spence C. (2022). Exploring group differences in the crossmodal correspondences. Multisensory Research, 35, 495–536. https://doi.org/h79s 35985650 10.1163/22134808-bja10079

[bibr96-20416695221127325] Spence C. Deroy O. (2014). On the shapes of flavours: A review of four hypotheses. Theoria et Historia Scientiarum, 10, 207–238. https://doi.org/hjk3

[bibr97-20416695221127325] Spence C. Levitan C. A. (2021). Explaining crossmodal correspondences between colours and tastes. i-Perception, 12(3), 1–28. https://doi.org/hgtx 10.1177/20416695211018223 PMC8216361 34211685

[bibr98-20416695221127325] Spence C. Levitan C. A. (2022). Exploring the links between colours and tastes/flavours. Journal of Perceptual Imaging, 5, 000408-1–000408-16. 10.2352/J.Percept.Imaging.2022.5.000408

[bibr99-20416695221127325] Spence C. Ngo M. (2012). Assessing the shape symbolism of the taste, flavour, and texture of foods and beverages. Flavour, 1, Article 12. 10.1186/2044-7248-1-12

[bibr100-20416695221127325] Spence C. Wan X. Woods A. Velasco C. Deng J. Youssef J. Deroy O. (2015). On tasty colours and colourful tastes? Assessing, explaining, and utilising crossmodal correspondences between colours and basic tastes. Flavour, 4(23), 1–17. 10.1186/s13411-015-0033-1

[bibr101-20416695221127325] Spence C. Youssef J. (2019). Synaesthesia: The multisensory dining experience. International Journal of Gastronomy and Food Science, 18, Article 100179. 10.1016/j.ijgfs.2019.100179

[bibr102-20416695221127325] Stewart P. C. Goss E. (2013). Plate shape and colour interact to influence taste and quality judgments. Flavour, 2, Article 27. 10.1186/2044-7248-2-27

[bibr103-20416695221127325] Sugimori E. Kawasaki Y. (2022). Cross-modal correspondence between visual information and taste perception of bitter foods and drinks. Food Quality and Preference, 98, Article 104539. https://doi.org/hwzk

[bibr104-20416695221127325] Sumner P. Mollon J. D. (2003). Did primate trichromacy evolve for frugivory or folivory? In Mollon J. D. Pokorny J. Knoblauch K. (Eds.), Normal and defective colour vision (pp. 21–30). Oxford University Press. 10.1093/acprof:oso/9780198525301.003.0003

[bibr105-20416695221127325] Sundar A. Noseworthy T. J. (2016). Too exciting to fail, too sincere to succeed: The effects of brand personality on sensory disconfirmation. Journal of Consumer Research, 43(1), 44–67. 10.1093/jcr/ucw003

[bibr106-20416695221127325] Trofimova I. (1999). An investigation of how people of different age, sex, and temperament estimate the world. Psychological Reports, 85(2), 533–552. 10.2466/pr0.1999.85.2.533 10611787

[bibr107-20416695221127325] Turoman N. Velasco C. Chen Y.-C. Huang P.-C. Spence C. (2018). Symmetry and its role in the crossmodal correspondence between shape and taste. Attention, Perception, & Psychophysics, 80(3), 738–751. 10.3758/s13414-017-1463-x 29260503

[bibr108-20416695221127325] Urushihara K. Miller R. R. (2009). Stimulus competition between a discrete cue and a training context: Cue competition does not result from the division of a limited resource. Journal of Experimental Psychology: Animal Behavior Processes, 35(2), 197–211. 10.1037/a0013763 19364229 PMC2844246

[bibr109-20416695221127325] Valdez P. Mehrabian A. (1994). Effects of color on emotions. Journal of Experimental Psychology: General, 123(4), 394–409. https://doi.org/drn57z 7996122 10.1037//0096-3445.123.4.394

[bibr110-20416695221127325] Velasco C. Hyndman S. Spence C. (2018a). The role of typeface curvilinearity on taste expectations and perception. International Journal of Gastronomy and Food Science, 11, 63–74. 10.1016/j.ijgfs.2017.11.007

[bibr111-20416695221127325] Velasco C. Michel C. Youssef J. Gamez X. Cheok A. D. Spence C. (2016). Colour–taste correspondences: Designing food experiences to meet expectations or to surprise. International Journal of Food Design, 1(2), 83–102. 10.1386/ijfd.1.2.83_1

[bibr112-20416695221127325] Velasco C. Salgado-Montejo A. Marmolejo-Ramos F. Spence C. (2014). Predictive packaging design: Tasting shapes, typefaces, names, and sounds. Food Quality and Preference, 34, 88–95. https://doi.org/hqg3

[bibr113-20416695221127325] Velasco C. Woods A. T. Deroy O. Spence C. (2015a). Hedonic mediation of the crossmodal correspondence between taste and shape. Food Quality and Preference, 41, 151–158. 10.1016/j.foodqual.2014.11.010

[bibr114-20416695221127325] Velasco C. Woods A. T. Hyndman S. Spence C. (2015b). The taste of typeface. i-Perception, 6(4), 1–10. 10.1177/2041669515593040 27433316 PMC4934647

[bibr115-20416695221127325] Velasco C. Woods A. Liu J. Spence C. (2016a). Assessing the role of taste intensity and hedonics in taste–shape correspondences. Multisensory Research, 29(1–3), 209–221. 10.1163/22134808-00002489 27311297

[bibr116-20416695221127325] Velasco C. Woods A. T. Marks L. E. Cheok A. D. Spence C. (2016b). The semantic basis of taste-shape associations. PeerJ, 4, Article e1644. 10.7717/peerj.1644 PMC4783761 26966646

[bibr117-20416695221127325] Velasco C. Woods A. T. Petit O. Cheok A. D. Spence C. (2016c). Crossmodal correspondences between taste and shape, and their implications for product packaging: A review. Food Quality and Preference, 52, 17–26. 10.1016/j.foodqual.2016.03.005

[bibr118-20416695221127325] Velasco C. Woods A. T. Wan X. Salgado-Montejo A. Bernal-Torres C. Cheok A. D. Spence C. (2018b). The taste of typefaces in different countries and languages. Psychology of Aesthetics, Creativity, and the Arts, 12(2), 236–248. 10.1037/aca0000120

[bibr119-20416695221127325] Vogel T. Ingendahl M. Winkielman P. (2021). The architecture of prototype preferences: Typicality, fluency, and valence. Journal of Experimental Psychology: General, 150(1), 187–194. 10.1037/xge0000798 32584126

[bibr120-20416695221127325] Wan X. Velasco C. Michel C. Mu B. Woods A. T. Spence C. (2014a). Does the type of receptacle influence the crossmodal association between colour and flavour? A cross-cultural comparison. Flavour, 3(1), Article 3. 10.1186/2044-7248-3-3

[bibr121-20416695221127325] Wan X. Woods A. T. van den Bosch J. J. F., McKenzie K. J., Velasco C. Spence C. (2014b). Cross-cultural differences in crossmodal correspondences between basic tastes and visual features. Frontiers in Psychology, 5, Article 1365. https://doi.org/ghj59v 10.3389/fpsyg.2014.01365 PMC4259000 25538643

[bibr122-20416695221127325] Wang Q. J. Reinoso Carvalho F. Persoone D. Spence C. (2017). Assessing the effect of shape on the evaluation of expected and actual chocolate flavour. Flavour, 6(1), Article 2. 10.1186/s13411-017-0052-1

[bibr123-20416695221127325] Wang Q. J. Wang S. Spence C. (2016). “Turn up the taste”: Assessing the role of taste intensity and emotion in mediating crossmodal correspondences between basic tastes and pitch. Chemical Senses, 41(4), 345–356. 10.1093/chemse/bjw007 26873934 PMC4840871

[bibr124-20416695221127325] Weierich M. R. Wright C. I. Negreira A. Dickerson B. C. Barrett L. F. (2010). Novelty as a dimension in the affective brain. NeuroImage, 49(3), 2871–2878. 10.1016/j.neuroimage.2009.09.047 19796697 PMC2818231

[bibr125-20416695221127325] Wendt D. (1968). Semantic differentials of typefaces as a method of congeniality research. Journal of Typographic Research, 2(1), 3–25. https://journals.uc.edu/index.php/vl/article/view/5016

[bibr126-20416695221127325] Whiteford K. L. Schloss K. B. Helwig N. E. Palmer S. E. (2018). Color, music, and emotion: Bach to the blues. i-Perception, 9(6), 1–27. 10.1177/2041669518808535 PMC6240980 30479734

[bibr127-20416695221127325] Wilson B. (2008). Swindled: From poison sweets to counterfeit coffee: The dark history of the food cheats. John Murray.

[bibr128-20416695221127325] Winkielman P. Ziembowicz M. Nowak A. (2015). The coherent and fluent mind: How unified consciousness is constructed from cross-modal inputs via integrated processing experiences. Frontiers in Psychology, 6, Article 83. 10.3389/fpsyg.2015.00083 PMC4327174 25741297

[bibr129-20416695221127325] Woods A. T. Marmolejo-Ramos F. Velasco C. Spence C. (2016). Using single colors and color pairs to communicate basic tastes II: Foreground–background color combinations. i-Perception, 7(5), 1–20. https://doi.org/f97s2x 10.1177/2041669516663750 PMC5034334 27708752

[bibr130-20416695221127325] Woods A. T. Spence C. Butcher N. Deroy O. (2013). Fast lemons and sour boulders: Testing crossmodal correspondences using an internet-based testing methodology. i-Perception, 4(6), 365–379. 10.1068/i0586 24349696 PMC3859554

[bibr131-20416695221127325] Wu Y. Jiang Y. (2022). Happy sweets or not? The affective effect of different concentrations of sweetness. *IVSP 2022: 2022 4th International Conference on Image, Video and Signal Processing*, 179–183. https://doi.org/hxdq

[bibr132-20416695221127325] Yang Q. Kraft M. Shen Y. MacFie H. Ford R. (2019). Sweet liking status and PROP taster status impact emotional response to sweetened beverage. Food Quality and Preference, 75, 133–144. https://doi.org/gntsxb

